# Bridging spatial scales in respiratory viral infection

**DOI:** 10.1073/pnas.2614436123

**Published:** 2026-10-20

**Authors:** Ulrik Hvid, Bjarke Frost Nielsen, Kim Sneppen

**Affiliations:** ^a^PandemiX - Center for Interdisciplinary Study of Pandemic Signatures, Copenhagen 2300, Denmark; ^b^ Biocomplexity, Niels Bohr Institute, University of Copenhagen, Copenhagen 2100, Denmark

**Keywords:** within-host modeling, SARS-CoV-2, influenza A, dynamical models, host–pathogen interactions

## Abstract

Respiratory viruses spread within the host through a combination of local expansion and occasional long-range dissemination that seeds new infection foci. We present LEAP (Lotka-Euler Airway Pathogen model), an analytically tractable within-host model that captures this two-scale process. The model takes the form of an age-dependent branching process and yields a closed-form expression for the exponential growth rate during early infection. Using empirical data to parameterize the model, we find that productive dissemination requires only a small number of successful long-range seeding events per infected cell, with distinct values for SARS-CoV-2 and influenza A virus. LEAP further predicts that, in these well-adapted viruses, interferon-mediated restriction only weakly affects exponential growth, while remaining decisive for poorly adapted pathogens. More broadly, the model provides a flexible framework for experimentally testable predictions of early infection dynamics.

## Significance

Respiratory viral infection is an inherently spatial process, in which the virus must colonize large areas of the airways to optimize reproduction. Recent studies in animal models infected with influenza A or SARS-CoV-2 have documented long-range stochastic jumps of viral populations between distant regions of the respiratory tract. The emerging picture is one of two co-occurring spatial processes: localized expansion of infected patches and long-range seeding events that are rare but crucial to rapid colonization of the airways. We introduce LEAP (Lotka-Euler Airway Pathogen model), a simple mathematical within-host model that bridges these two spatial scales. Using LEAP, measurements from the petri dish can directly produce predictions of infection dynamics in the body.

During viral infection of the respiratory system, the virus requires continual access to uninfected, susceptible cells. If a virion is released in an area of the lung with a high concentration of virus particles and where, perhaps, antiviral signaling has begun to limit susceptibility, the virus must move to another part of the respiratory tract (RT) to reproduce. Rapid dissemination will maximize viral shedding of the host, increasing the probability of infecting other individuals (1). Thus, viral colonization of the RT is a spatial process in which the virus must spread as widely as possible in the time window preceding activation of a systemic immune reaction involving widespread inflammation and recruitment of leukocytes (2). In the early stages of a successful respiratory infection, viral load grows exponentially in the host (3–6). In vitro cell culture studies can, however, typically only show local neighbor-to-neighbor spreading of viruses (7), and such spatially constrained spread is incompatible with exponential growth. Although in vitro studies with liquid overlays can exhibit diffuse plaques and enhanced seeding of secondary plaques (7), this is still a far cry from the long-range transport observed in vivo. Indeed, recent studies (8–10) have made it clear that in vivo infection involves long-distance transport of viral particles, facilitated by air or bloodstream. Most notably, a recent study in PNAS clearly documented that infection with the H1N1 influenza A virus (IAV) in ferrets involves long-range stochastic jumps between regions of the respiratory system (8). Similarly, it has been shown that, in mice coinfected with IAV strains that differ only by a fluorescent reporter protein, discrete islands of infection are established in the lungs (9, 10). In infected hamsters, SARS-CoV-2 establishes separate infection nodes throughout the RT (11).

Thus, the dynamics of viral proliferation involve an interplay between local spread among neighboring cells and long-range jumps of viral particles that can initiate new foci of infection (1). These observations provide clues to models that may reconcile the spatially constrained growth observed in vitro with the observations from human challenge studies, which show exponential growth during the first 2 to 4 d of viral infection. For SARS-CoV-2, viral load has been shown to double every ≈2 to 3 h in immune-naïve humans, peaking at 6.5 d postinfection (dpi) (3, 4). With IAV (H3N2) in humans, viral load can increase by 3 orders of magnitude between day 1 and 2 postinfection (12), corresponding to a similar doubling time to SARS-CoV-2, and peaking at 3 dpi.

We can thus think of in vivo infection as progressing at two separate spatial scales. The majority of published in-host dynamical infection models fall into two categories, each effectively describing one of these scales. Target-cell limited (TCL) models consist of Lotka–Volterra-style ordinary differential equations that treat the body as one well-mixed container, or a small number of organs that individually are well mixed (13). Over the past three decades, such models have achieved considerable success in elucidating fundamental aspects of viral kinetics (14–19). In particular, landmark studies of HIV dynamics have shed light on key quantities such as the lifespans of productively and nonproductively infected cells, the effects of (combination) antiretroviral therapy and the multiple phases of decline in plasma viremia associated with treatment (20). These achievements demonstrated how even highly idealized compartmental models—when combined with clinical and in vitro data—can yield important insights. Despite these successes, classical compartmental models relying on the assumption of homogeneous mixing often lack the granularity to discriminate between mechanisms. Furthermore, the acute diseases considered in this paper are fast-acting relative to HIV, making the well-mixed assumption less appropriate in this context. Another class of models have explicitly represented space, at the cost of being limited to describing local dissemination of viruses between neighboring cells (21–26). These models generally struggle to produce long periods of exponential growth and are typically not analytically tractable.

In this study, we present a modeling framework that bridges the two spatial scales described above and reliably yields exponential growth (or decay), at a rate which may be determined analytically from few parameters. We call it the Lotka-Euler Airway Pathogen model or LEAP (Lotka-Euler Airway Pathogen model). Like TCL models, LEAP is analytically tractable, but allows for a more realistic representation of viral propagation, using a quasi-spatial representation. LEAP can take parameters directly from in vitro measurements, going beyond a phenomenological description that risks overfitting. We demonstrate a few simple extensions that increase biological realism while maintaining analytical clarity. With these extensions, which include an eclipse phase and effects of interferon, we estimate the model parameter that is most difficult to measure directly, namely the average number q of virions secreted by an infected cell that successfully initiate new foci elsewhere. We estimate this “jump parameter,” for both SARS-CoV-2 and IAV. LEAP also allows for numerical, rather than analytical, inputs, enabling parameterization from experimental data or detailed numerical simulations. We give an example of this in *SI Appendix*, section C.

## Results

### The Minimal Model.

First, we present the simplest and most idealized version of LEAP. As we will show later, the minimal model has important limitations in terms of biological realism, but it illustrates the central concepts of this study. We represent local spread as a circular viral plaque or infection focus that grows at a constant radial speed v, as has been observed in cell cultures (27–29). These studies suggest a constant speed during the plaque growth phase of the order of 10 to 20 $\mu \text{m}/\text{h}\approx 1\;d_{c}/\text{h}$, where $d_{c}$ is the typical diameter of an epithelial tissue cell. Virions released within the locally growing focus will then have a chance to be transported, via air or bloodstream, to another location in the RT. That new focus is assumed to be far enough away that the circles never overlap. The area A of a focus grows with its age a as

[1] $$\begin{array}{c} A\left(a\right)=\pi {\left(v\;a\right)}^{2} \end{array}$$

We use spatial units of $d_{c}$ such that the area has units ${d_{c}}^{2}$ and simultaneously represents the number of infected cells. We use q to denote the jump parameter, defined as the number of new foci seeded per infected cell. Dimensionally, q is not a number per cell, but per unit area ${d_{c}}^{2}$. However, we assume a confluent monolayer with cell number density $1/{d_{c}}^{2}$. This is clearly a natural assumption in a monolayer culture, but justifiable even in vivo, since many common respiratory viruses predominantly infect and reproduce in the ciliated top layer of the epithelium (30–33). Regardless of whether the jumping virions do so individually or in groups, q is the number of foci seeded at a distant location per infected cell. *En bloc* transport of groups of virions has been documented for several species of viruses (34), and this mode would conceivably be of benefit to IAV, whose virions are often unable to infect in isolation (34, 35). For simplicity, we will describe jumping viruses as solitary, but this has no effect on the dynamics.

The rate of new infections in a focus is the time-derivative of the area A, and the rate of production of new foci [seeding rate b(a)] is

[2] $$\begin{array}{c} b\left(a\right)=q\cdot \frac{\text{d}A}{\text{d}a}=2\pi qv^{2}a=k^{2}\cdot a, \end{array}$$

[3] $$\begin{array}{c} k\equiv \sqrt{2\pi q}v. \end{array}$$

The simplifying assumption here is that all viruses are released from the very edge of the plaque and cells behind the front have died. In fact, both IAV and SARS-CoV-2 virions shed from an infected cell for several hours before the cell dies (36, 37). In *SI Appendix*, section C, we present a more detailed numerical model, taking finite shedding periods into account. While this has no effect on the qualitative conclusions, it does change the estimated q-value somewhat (Table 1).

**Table 1.** Model parameters for SARS-CoV-2 and IAV

Parameter	Value	Unit	Source
SARS-CoV-2			
v	1	$d_{c}$/h	(38)
$a_{g}$	16	h	*SI Appendix*, Fig. S1
p	$10^{-4}$	$d_{c}^{-2}$ (cell^−1^)	(38)
λ	0.25	h^−1^	(3, 4)
q	0.5 (0.7)	$d_{c}^{-2}$ (cell^−1^)	Analytical (CA) est.
Influenza A			
v	1	$d_{c}$/h	(27)
$a_{g}$	6	h	(36)
p	$50\cdot 10^{-4}$	$d_{c}^{-2}$ (cell^−1^)	(39)
λ	0.25	h^−1^	(12)
q	0.07 (0.2)	$d_{c}^{-2}$ (cell^−1^)	Analytical (CA) est.

**Fig. 1.**
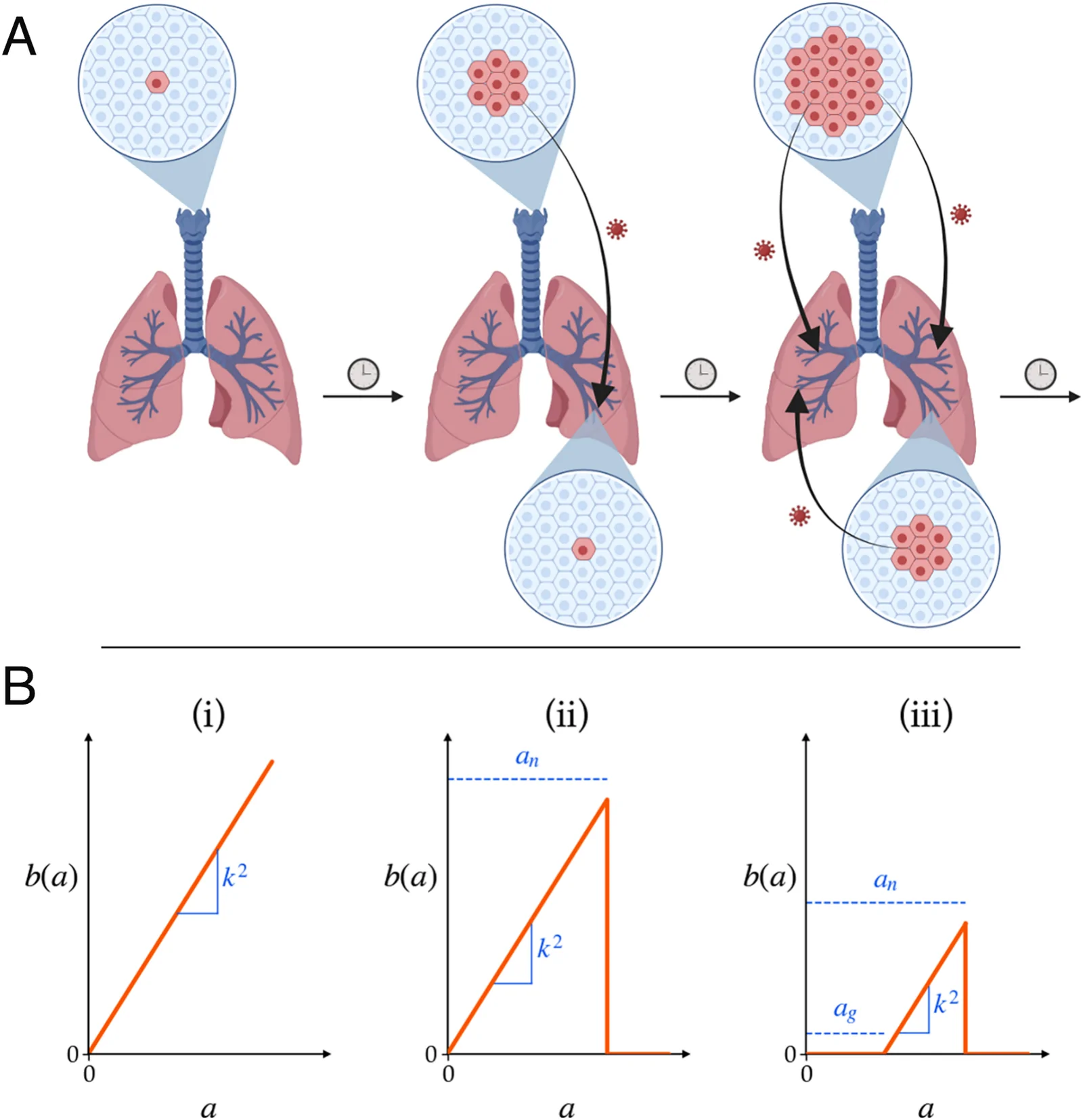
Model of the interplay of short- and long-range dynamics. (*A*) The model consists of foci of infection that expand and replicate. Local expansion (red) happens at a constant radial speed. Each newly infected cell seeds q (typically <1) new foci of infection. Made with BioRender (40). (*B*) The three different reproduction kernels used. (*i*) Minimal model. Linear growth starting at age a=0 and growing without bounds. (*ii*) First extension. Seeds go discontinuously to zero at $a=a_{n}$. The actual input parameter is p, while $a_{n}$ is the age that satisfies $A(a_{n})=1/p$ (Eq. **12**). (*iii*) In the final extension, we introduce an eclipse phase $a_{g}$ between the seeding of a new focus and the time when it starts producing offspring. In all cases, $k=\sqrt{2\pi q}v$.

The system represents an age-dependent birth process in which the seeding rate b(a) of each individual is proportional to its age. The analysis can then proceed without explicitly considering expanding circles, but instead taking the individual growing focus as the reproductive unit. We call b(a) the reproduction kernel and it contains many of the biological assumptions made in the model. In this study, we will also analyze more realistic reproduction kernels (Fig. 1*B*) as well as a numerically determined kernel from a detailed cellular automaton model of an infection focus (*SI Appendix*, section C).

From the kernel, we can derive the time evolution of the system as a whole. We will solve the simplest model (*i*) (Fig. 1*B*) in the main text and refer to *Materials and Methods* for the more involved derivations of models (*ii*) and (*iii*). Define B(t) as the total rate of seeding in the system at time t. Then B(t−a)b(a) is the rate of seeds contributed by individuals of age a. Since B(t) is the sum of all these contributions, plus the seeding rate b(t) of the initial focus,

[4] $$\begin{array}{c} B\left(t\right)=b\left(t\right)+\int_{0}^{t}B\left(t-a\right)\;b\left(a\right)\;\;\text{d}a=b\left(t\right)+\left(B*b\right)\left(t\right), \end{array}$$

where ∗ is the Volterra convolution (41). We then take the Laplace transform, which converts convolutions to products (42):

[5] $$\begin{array}{c} \hat{B}\left(\lambda \right)=\hat{B}\left(\lambda \right)\cdot \hat{b}\left(\lambda \right)+\hat{b}\left(\lambda \right)\Rightarrow \hat{B}\left(\lambda \right)=\frac{\hat{b}\left(\lambda \right)}{1-\hat{b}\left(\lambda \right)}. \end{array}$$

To recover B(t), we look for poles of the Laplace transform (43). In this case, a pole is a value λ that satisfies $\hat{b}\left(\lambda \right)=1$, or

[6] $$\begin{array}{c} \hat{b}\left(\lambda \right)=\int_{0}^{\infty }\;e^{-\lambda a}\;b\left(a\right)\;\;\text{d}a=1. \end{array}$$

Eq. **6** is a version of the Lotka-Euler (LE) equation, which is commonly used to describe population growth when reproduction is age-dependent (44, 45). Using the kernel from Eqs. **2** and **6** becomes

[7] $$\begin{array}{c} \hat{b}\left(\lambda \right)=\frac{k^{2}}{\lambda^{2}}=1\Rightarrow \lambda =\pm k=\pm v\sqrt{2\pi q}, \end{array}$$

Since the positive growth rate dominates in the long run, we ignore the decaying λ<0 solution. In conclusion,

[8] $$\begin{array}{c} B\left(t\right)\propto e^{\mathrm{kt}}. \end{array}$$

An exact derivation would yield B(t)=λsinh(λt), but the procedure above—finding the largest λ solving the LE equation [the dominant pole of $\hat{B}\left(\lambda \right)$ (43)] and assuming exponential growth—is easily generalizable to more complicated kernels and correctly identifies the growth rate that dominates after a transient. We assume $\int_{0}^{\infty }b\left(a\right)\;\;\text{d}a>1$ in all cases, since this is required for a growing population and ensures the existence of a positive λ.

After the transient, the population of foci settles into an exponential age distribution

[9] $$\begin{array}{c} P\left(a\right)=\lambda \;e^{-\lambda a} \end{array}$$

yielding the mean values of age and area, respectively,

[10] $$\begin{array}{c} \left\langle a\right\rangle =\int_{0}^{\infty }a\;P\left(a\right)\;\;\text{d}a=\frac{1}{\lambda } \end{array}$$

**Fig. 2.**
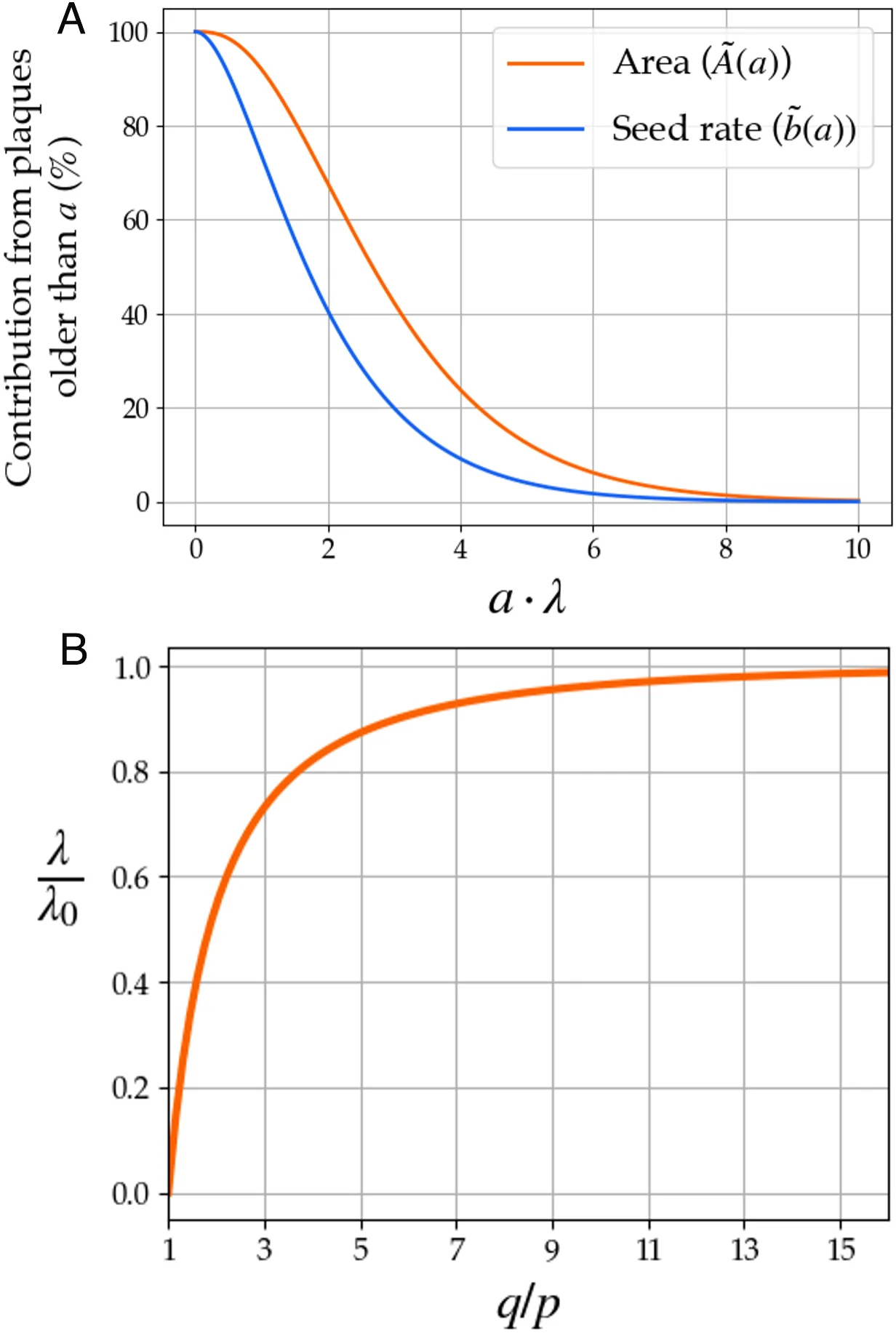
Diminishing returns from cytokine signaling in limiting viral growth rate. (*A*) Survival function of $\tilde{A}$ (orange) and $\tilde{b}$ (blue). $\tilde{A}\left(a\right)\equiv P\left(a\right)\cdot A\left(a\right)$ and $\tilde{b}\left(a\right)\equiv P\left(a\right)\cdot b\left(a\right)$ are the relative contribution by foci of age a to the total infected area and total seeding rate, respectively. We see that foci with a>6/λ contribute less than 10% of total infected area and ≈1% of new seeds. (*B*) Effect of IFN signaling on growth rate, measured as the actual growth rate λ divided by the counterfactual growth rate $\lambda_{0}$ in the absence of IFN. The asymptotic shape shows that, if p≪q, the virus cannot increase growth rate further by decreasing p.

[11] $$\begin{array}{c} \left\langle A\right\rangle =\int_{0}^{\infty }\pi {\left(v\cdot a\right)}^{2}\;P\left(a\right)\;\;\text{d}a=\int_{0}^{\infty }\tilde{A}\left(a\right)\;\;\text{d}a=\frac{1}{q}, \end{array}$$

using Eq. **7**. Here, $\tilde{A}\left(a\right)\equiv A\left(a\right)\cdot P\left(a\right)$ is the contribution to the mean area due to foci of age a, and it is plotted in Fig. 2*A*. Note that none of these results require knowledge of the viral generation time, the burst size of an infected cell, or the diffusive properties of viral particles in epithelial tissue. Instead, these properties combine into the emergent expansion speed v, which can be estimated from in vitro measurements.

### Introducing IFN Response.

The LE equation typically includes a death factor (45, 46) that brings the seeding rate down to zero with increasing age, but we have so far allowed b(a) to grow unbounded. As such, the reproduction number of a focus is infinite in the minimal model. Intuitively, one might guess that this implies superexponential growth, but the analysis above shows that this is not the case. The reason is that the significance of older foci gets “diluted” among the much greater number of younger offspring (Fig. 2*A*).

Still, real infection foci do not grow infinitely, and we should examine how problematic this assumption is. The first extension of the model [(*ii*), Fig. 1*B*] has foci stop their growth due to the first line of immune defense, namely cytokine signaling and the resulting antiviral state in infected tissue cells. It is well documented that interferon (IFN)-competence in host cells can have the effect of halting expansion of viral plaques (24, 47), and in differentiated air–liquid interface (ALI) cultures of primary human bronchial epithelial cells infected with SARS-CoV-2, spatial expansion stops after ⪅3 d (38). Mutant viruses lacking a mechanism for antagonizing IFN-response produce smaller plaques than their wild-type counterparts (48). In this way, the expected final size of a plaque is related to the fraction p of infected cells that secrete IFN. We make the simplifying assumption that once a single cell has secreted IFN, this immediately halts the growth of the infection focus. This is motivated by the well-documented paracrine feedback loop in which IFN stimulation in turn increases the probability of IFN secretion (49–52), resulting in a signaling cascade. For mathematical simplicity, we further model this process as deterministic, assuming that focus expansion always stops when the area is exactly 1/p. The result is the kernel (*ii*) shown in Fig. 1*B*. This gives a clear qualitative description of the host’s first line of defense, albeit highly idealized. In *SI Appendix*, we soften these assumptions, first analytically (*SI Appendix*, section B), and then in a cellular automaton (CA) simulation (*SI Appendix*, section C), and find that our results are relatively insensitive to this particular idealization.

The probability P that an infected cell secretes IFN and stops expansion of the local infection focus is pathogen-specific. An order-of-magnitude estimate of P for SARS-CoV-2 can be inferred from experiments in ref. 38, since in those cultures infection typically stops after ≈10^4^ infected cells, suggesting $P\approx 10^{-4}$. For IAV, which is far less competent at antagonizing antiviral responses (53), P has been estimated 50 times higher, $5\cdot 10^{-3}$ (39). According to Eq. **1**, A(a)=1/p when

[12] $$\begin{array}{c} a=a_{n}\equiv \frac{1}{v\sqrt{\pi p}}. \end{array}$$

Now, $b\left(a\right)=k^{2}\cdot a$ for $a<a_{n}$ and b(a)=0 for $a>a_{n}$. The growth rate is found by solving the LE equation (Eq. **6**) but taking the integral only up to $a_{n}$. The solution is the transcendental equation

[13] $$\begin{array}{c} 1-\left(1+\lambda a_{n}\right)e^{-\lambda a_{n}}=\frac{\lambda^{2}}{k^{2}}. \end{array}$$

This equation shows the dependence of the growth rate on $a_{n}$ and thus P. When $a_{n}\to \infty$, we recover Eq. **7**. To evaluate the effect of IFN signaling on growth rate, we define

[14] $$\begin{array}{c} \lambda_{0}\left(v,q\right)=\lambda \left(v,q;\;p=0\right), \end{array}$$

i.e. the growth rate that would be observed in an equivalent system with no IFN response. Then we can quantify the effect of IFN response by $\lambda /\lambda_{0}$, as in Fig. 2*B*. We find a steeply rising $\lambda /\lambda_{0}$ for q/p≈1 beyond which it asymptotes to 1. Evidently, decreasing p by antagonizing antiviral signaling yields diminishing returns for the virus. In general, if p⪅q/10, a further reduction in p hardly affects the growth rate (Fig. 2*B*). The reason is apparent from Fig. 2*A*; although the oldest foci are, individually, the largest and most productive, the exponential age distribution (Eq. **9**) implies that they are much less abundant than the younger foci. As a result, the total infected area and the seeding rate are dominated by younger, smaller foci with age a<10/λ. This means that unless IFN kicks in at $a_{n}\ll 10/\lambda$, the effect on growth dynamics will be negligible.

### The Full Model—Introducing Eclipse Phase.

In terms of biological realism, the main drawback of the models presented so far is the fact that a newly seeded focus starts producing offspring immediately. This is problematic, because the time it takes for the seed cell to even start transcribing viral genes on a large scale is greater than the doubling time of the system, e.g., ≈6 h for SARS-CoV-2 (54) vs. 2 to 3 h of doubling time (3, 4). Knowing this, the result from Fig. 2*A* that foci with a<1.5/λ≈6 h account for 50% of the seeding rate cannot be remotely correct. Therefore, we introduce an eclipse time $a_{g}$ in the growth of each focus, leading to the reproduction kernel (*iii*) shown in Fig. 1*B*. The mathematics of this full model is derived in *Materials and Methods*, where we show that the dynamics are fully contained in the equations

[15] $$\begin{array}{c} r^{2}=e^{(1-r)\alpha }\left(1-e^{-\beta r}\left(1+\beta r\right)\right), \end{array}$$

**Fig. 3.**
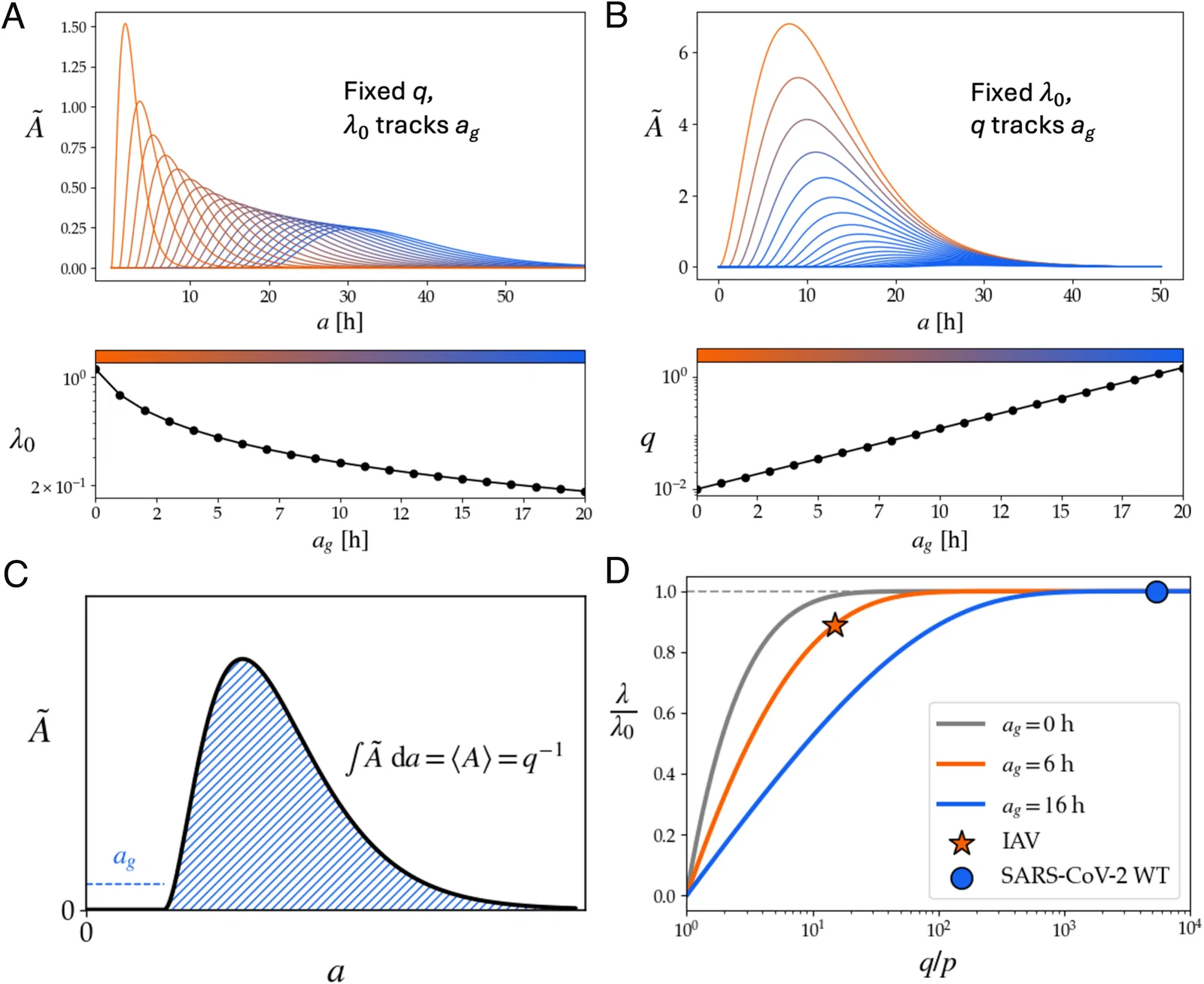
Introducing eclipse phase in local growth alters dynamics. (*A*) Distributions of the age-dependent area contribution $\tilde{A}\left(a\right)$ for various values of the eclipse time $a_{g}$ and fixed propagation speed v=1 and jump parameter q=0.2. Increasing $a_{g}$ decreases $\lambda_{0}$ according to Eq. **16**. (*B*) When keeping $\lambda_{0}$ fixed by adjusting q, the area under the curve shrinks as q increases according to Eq. **28**. Note that q must increase exponentially with $a_{g}$ to ensure a constant $\lambda_{0}$. Here, $v=1,\lambda_{0}=0.25$. (*C*) Schematic of the relation between the $\tilde{A}$ distribution, the mean area, and the jump parameter. As shown in *SI Appendix*, section A, the area under the curve is equal to $q^{-1}$. (*D*) Effects of IFN on the exponential growth rate. The effect is quantified by $\lambda /\lambda_{0}$, the ratio of the actual growth rate to the growth rate that would be observed in the absence of IFN signaling.

[16] $$\begin{array}{c} \lambda_{0}=\frac{2}{a_{g}}W\left(\frac{a_{g}}{2}k\right)\;\mathrm{if}\;a_{g}>0,k\;\mathrm{if}\;a_{g}=0. \end{array}$$

with $r\equiv \lambda /\lambda_{0},\alpha \equiv a_{g}\lambda_{0},\;\beta \equiv \left(a_{n}-a_{g}\right)\lambda_{0}$ and W(·) the Lambert W function (55). Fig. 3 shows how the dynamics depend on $a_{g}$. If we fix q and allow $\lambda_{0}$ to change according to Eq. **16**, we get Fig. 3*A*. We see that $a_{g}$ decreases the growth rate, which has the effect of widening the $\tilde{A}$-distribution, increasing the contribution of older and larger foci. Interestingly, even in the full model, $\left\langle A\right\rangle =\int \tilde{A}\;da=q^{-1}$, so the area under the curve remains constant (Fig. 3*C*, proven in *SI Appendix*, section A).

Instead of keeping q fixed, we can also let it track $a_{g}$ to keep $\lambda_{0}$ constant. The result is shown in Fig. 3*B*, where $\lambda_{0}=0.25$ h^−1^. The *Bottom* panel shows that this requires q to increase exponentially with $a_{g}$ (Eq. **28**). The increasing value of q is expressed as a decreasing area under the $\tilde{A}$-curve as $a_{g}$ increases, according to Eq. **28**. As in model (*ii*), the virus gets diminishing returns from decreasing p, but Fig. 3*D* shows that the domain over which IFN has a measurable influence now increases with $a_{g}$. In *SI Appendix*, section D we show that this dependence is approximately exponential, with IFN being ineffectual for $q/p⪆e^{\lambda_{0}a_{g}}$.

### Estimating the Jump Parameter.

To estimate q, we need to fix all other parameters, as well as the resulting growth-rate λ. The parameters for SARS-CoV-2 and IAV are shown in Table 1. To determine $a_{g}$, we looked at one-step growth curves, i.e., viral titers from cultures infected at high multiplicity of infection, to represent the output of just one viral generation. We set $a_{g}$ equal to the time when the viral output of the seed-cell has the biggest impact on overall growth. For SARS-CoV-2 the curve has a complex shape, with a small output already at ≈6 hpi, but increasing by many orders of magnitude over the next 36 h (37). To determine the time at which the output of the seed cell is most significant for growth, we discount the late offspring with the factor $e^{-\lambda t}$—in line with the logic of the LE equation—and locate the peak (*SI Appendix*, Fig. S1). For the wild-type (WT) variant, there is a clear peak at 16 hpi, and for Delta there is a peak at 12 hpi. Since the exponential growth rate was measured for WT SARS-CoV-2 (3, 4), we use 16 hpi. For IAV, the curve is simpler, as the shedding rate increases almost stepwise at around 6 hpi (36), so we set $a_{g}=6$ h for IAV.

To determine q, we solve Eq. **27**. We get $q=0.5\;{d_{c}}^{-2}$ for SARS-CoV-2 WT. As a sanity check, the time $a_{n}$ it takes for an infection focus to reach $A(a_{n})=1/p$ and stop growing is 72 h in agreement with the ≈3 d it takes for a plaque to stop growing in ALI cultures (38). For IAV, we get q=0.07. The fact that the jump rate is much lower for IAV may be related to the well-known phenomenon that individual IAV particles are typically not able to complete infection alone (35, 56). One of the reasons is that the majority have deleterious mutations (39). In addition, these mutations are strongly correlated with increased activation of antiviral responses, contributing to the higher p for IAV. Conceptually, the jump parameter can be decomposed into several independent factors:

[17] $$\begin{array}{c} q=n_{v}\cdot p_{j}\cdot p_{a}\cdot p_{r}, \end{array}$$

where $n_{v}$ is the number of infectious progeny per cell, $p_{j}$ is the probability that a virion is transported to a distant location in the RT and penetrates the mucus layer there, $p_{a}$ is the probability that such a virion adsorbs into a cell at that location, and $p_{r}$ is the probability of completing reproductive infection there. Then, the high fraction of defective IAV particles [70 to 99% (56)] would reduce q via $p_{r}$.

To appreciate the magnitude of q, it is useful to compare with a (highly idealized) well-mixed null model in which all infections are due to long-range jumps from a single cell (no local spread). Simplifying by taking a reproduction kernel $b\left(a\right)=q\delta \left(a-a_{0}\right)$ with $a_{0}$ the generation time and δ denoting Dirac’s delta, one would find $q=2^{a_{0}/T_{2}}$ with $T_{2}$ the doubling time. For a virus with a generation time of 6 h and a doubling time of 3 h, one would thus infer $q=4\;{d_{c}}^{-2}$. The estimate of $q=0.5\;{d_{c}}^{-2}$ for SARS-CoV-2 is thus far from this well-mixed limit, and IAV even more so.

In terms of IFN suppression, we predict that for IAV, p is in the regime of continuing returns, as opposed to SARS-CoV-2 (Fig. 3*D*). If p were doubled, this would reduce λ from 0.25 h^−1^ to 0.21 h^−1^, which compounds into a difference of a factor of ≈7 over 48 h.

In *SI Appendix*, section C we make a separate estimate of q using a numerical reproduction kernel derived from a cellular automaton (CA) model that relaxes the strongest assumptions built into the analytical kernels used here. These separate estimates are included in parentheses in Table 1. We find that, while the assumption of a deterministic and sudden halting of the plaque by IFN might seem to be the crudest assumption, its quantitative effect is in fact relatively small. Instead, the idealization that the infection focus abruptly sets into constant-speed growth, with no transient accelerating phase turns out to be more consequential. The reason is that, due to exponential discounting, approximations regarding the activity of older foci are much less consequential than those regarding early stages. The LE equation tells us that we need to know the early stages in great detail but are allowed some leniency when it comes to later stages.

## Discussion

Our analysis indicates that the early exponential growth rate of a respiratory infection is governed chiefly by the seeding of new foci and is largely indifferent to the fate of larger, established foci. This followed from the minimal version of the LEAP model, which couples local focus expansion with stochastic long-range seeding events. A single focus contributes new infections only through its growing perimeter, which expands linearly in time, whereas long-range seeding enables overall exponential growth. Any individual older focus is therefore rapidly dwarfed by the younger foci it has spawned.

Two refinements add further biological realism. A focus-level eclipse phase $a_{g}$ (Fig. 1*B*) removed the unphysical instantaneous shedding of foci. This strongly affected estimates of q, the number of jumping virions per infected cell (Fig. 3*B*). Including a cap on plaque size via p as well, due to effects of interferon, yielded q-estimates on the order of 0.5 for SARS-CoV-2 and 0.07 for IAV. Because large foci already contribute little, we find that suppressing IFN beyond a certain point yields vanishing returns. In the absence of an eclipse phase, we found that, below roughly p/q≈1/10, further suppression hardly changes the growth rate. Incorporating an eclipse phase $a_{g}$ shifts this estimate to lower p/q (*SI Appendix*, Eq. **S16**). In any case, we find that SARS-CoV-2 exists squarely in the regime of vanishing returns (Fig. 3*D*), while modest gains are available for IAV. This result may reflect the high degree of host-adaptation of these viruses.

In traditional target-cell-limited models, variables represent total scalar amounts of virus, infected/susceptible cells, IFN, etc. in the body. A major benefit of the LEAP model is that it also predicts the distribution of these quantities between different infection sites, enabling additional falsifiable predictions. This is exemplified by our prediction that IFN deficiency will not affect the exponential growth rate for SARS-CoV-2 and will only modestly affect that of IAV. The evidence from mouse models of SARS-CoV-2 is divided: One study (57) found minimal effect of IFN deficiency on viral load at 2 dpi, whereas another (58) reported a substantial difference. Adjudicating between these two results would require reparameterizing LEAP to mice and comparing only titers within the exponential phase, which may be shorter for mice than for humans, given their smaller lungs.

The factorization of q into different components (Eq. **17**), also points to falsifiable predictions. Factors $n_{v}$ (burst size), $p_{a}$ (probability of adsorption), and $p_{r}$ (probability of reproductive infection) are measurable quantities that can be tuned with genetic modification, and our model makes concrete predictions as to how such changes should affect the growth rate in vivo. The estimated q-values are tiny compared to estimated burst sizes for IAV (59) and SARS-CoV-2 (60), meaning that a vanishing fraction of offspring make a successful jump. It is interesting to consider whether the virus has an ideal distribution between viruses that stay in the same focus, contributing to local spread, and those that jump. At a newly seeded infection site, viruses infecting neighbor cells are probably highly effective, benefiting from Allee effects arising from the complementation and cooperation between coinfecting virions (35, 61). At later stages, local saturation of virus may result in wasted offspring, or may even inhibit infection through defective interfering particles (56, 62). A lone jumping virus may be very unlikely to complete a successful infection, and yet allotting some jumpers is indispensable to achieving exponential growth. Variations on the LEAP model could address the question of whether the virus is best served by maximizing its number of jumpers, or whether some optimal distribution exists. Though the jump parameter q is largely determined by the environment (the tissue in which the virus spreads), there are indications that the virus may also be able to adaptively adjust its q-value. For example, IAV has been shown to display receptor-binding hemagglutinin (HA) and receptor-cleaving neuraminidase (NA) proteins in a polarized fashion, facilitating their movement through sialic acid-rich host mucus (63, 64). In terms of the decomposition in Eq. **17**, this asymmetric protein organization increases $p_{j}$. Similarly, different viruses may respond differently to mucociliary clearance and even benefit from it at times (38).

At first glance, the result that the growth rate of SARS-CoV-2 is insensitive to the fraction of IFN-secreting cells P seems at odds with the well-established notion that the ability of the virus to interfere with immune signaling of host cells is the key to its success. SARS-CoV-2, in particular, is known to benefit from the ability to substantially delay IFN induction (53, 65, 66). However, there may be no contradiction here, as the molecular pathways leading to IFN secretion are simultaneously associated with the secretion of cytokines that activate the leukocytic immune system (67). The mouse experiments in ref. 57 showed precisely that, while viral load was unaffected by knocking out the receptor that binds type-I IFN, that mutation did significantly affect recruitment of inflammatory immune cells. Thus, while the virus’ returns on antagonizing IFN secretion may be negligible in terms of increasing its growth rate, it may still be able to extend its growth phase by offsetting the activation of a systemic innate immune response. SARS-CoV-2 titers typically peak later than those of IAV (4, 12), and this may be due precisely to its ability to extend IFN secretion from infected epithelial cells. Finally, the conclusion of diminishing returns from antagonizing IFN relates only to well-adapted viruses that already do this effectively. Any respiratory virus is subject to the condition that, to grow, it must have p<q, otherwise local growth will typically be halted before it can reproduce. It is plausible that IFN secretion from structural tissue continually prevents growth of viruses that are not adapted to humans but still enter our body. For instance, Newcastle Disease Virus (NDV)—a bird virus—triggers IFN secretion with as much as 25% probability (50) in mice, which probably renders successful infection of these mammals impossible. Indeed, knocking out IFN receptors in mouse macrophages drastically increases viral output of NDV (68).

While we have utilized piecewise linear reproduction kernels (as in Fig. 1), our framework can accommodate observed or numerically derived kernels directly. Explicit simulations using a lattice-based cellular automaton are able to represent details such as the finite shedding period of infected cells and the finite duration of the IFN-cascade that eventually halts the growth of the infection focus. The analysis presented in *SI Appendix*, section C shows that the most consequential simplification in the piecewise linear kernel is the immediate transition from no shedding to linear increase. In fact, this transition has a duration of a few viral replication cycles, during which the front establishes and shedding increases in a convex fashion. The numerical kernel derived in that analysis leads to somewhat higher inferred q values when subjected to the LEAP machinery.

### The Space-Free Assumption.

A few well-known models from the ecology literature bear mentioning. The minimal LEAP model (*i*), neglecting eclipse phase and IFN effects, is closely related to the scattered colony PDE model of Shigesada–Kawasaki–Takeda (69) with $\lambda \left(r\right)=\lambda_{1}r$, here using variables from that paper. However, in the LEAP framework, age a is uncoupled from radius r, which is what allows for later extensions that increase biological realism. In addition, the Lotka-Euler formulation highlights the inevitable convergence to exponential growth, which forces the discounting of late viral releases. As this study shows, that discounting is necessary to understand the effect of IFN on early-stage infection.

The two-scale simplicity of LEAP relies on the fact that it does not incorporate explicit jump distances, meaning that it can model biphasic spatial spread without introducing spatial coordinates. This is the core assumption of the model; other assumptions can be relaxed by changing the shape of the reproduction kernel and, if necessary, solving the LE equation numerically. The “space-free” assumption is an extremely useful idealization but is less accurate if most jumps are of intermediate distance, breaking the separation of scales. The spatial correlations observed in ref. 8 show that the dynamics are not entirely space-free, but it also clearly shows that very long jumps characterize the invasion of the lung. Some models from the ecology literature do explicitly represent space by utilizing spatial, rather than temporal, kernels (70, 71). For instance, deterministic integro-difference equation models with heavy-tailed kernels are able to produce geometric growth of an invading species (71). However, this result crucially relies on continuous variables and is not recovered by corresponding stochastic models (70, 72). With finite populations, extremely fat-tailed kernels, such as a power-law with divergent mean (γ<2), are required for faster-than-polynomial growth of the system (72). Such a model would imply two new dynamical effects. First, it would introduce coalescing foci, necessitating a higher q to achieve the same growth rate. Second, it would introduce an intermediate scale of distance at which effects related to multiplicity of infection (MOI) may play a role (35, 56). In our space-free model, it is reasonable to assume that two jumping virions never land on the same cell, implying that all jumps are independent. At intermediate distances, this assumption may not hold. In our opinion, however, a proper spatial representation of viral spreading in the lung requires modeling of the branching structure of the RT. Due to this branching, it is possible for two foci to be a short Euclidean distance from each other but still widely separated in terms of distance along the ciliated lung surface. In the future, we plan to explore the consequences of this structure on viral spread.

Regardless of the distribution of jump distances, the assumption of no overlap is increasingly violated as the amount of susceptible tissue decreases with infection. In a hamster infected with fluorescent SARS-CoV-2, the lungs appear nearly saturated already at 2 dpi (11). This raises the question of whether exponential growth is first curbed by immune responses or by lack of uninfected lung regions. In any case, the LEAP model claims only to describe the exponential growth phase when the fraction of infected or refractory cells in the RT is still vanishing.

In the RT, the activity of ciliated cells causes a directed upward flow of the mucus layer, and an infection focus is likely to spread anisotropically, along elongated structures. In vitro studies also show that the geometry of a plaque is affected by the viscosity of the overlay (analogously, the mucus layer) (7, 27–29). Still, an ALI study (38) of this motion indicates a characteristic speed similar to the one used in this analysis, and the precise geometry (expanding circle vs. expanding line) is not crucial for our analysis.

For virus types that shed their offspring from the live host cell over a period, e.g., IAV, SARS-CoV-2, or Vesicular stomatitis virus (73–75), the plaque expansion speed v is highly sensitive to the first viruses to leave an infected cell (76). For SARS-CoV-2, for example, infected cells can release some progeny virus as early as 6 hpi, but release the vast majority much later [*SI Appendix*, Fig. S1, (37)]. Possibly, this provides the strategic advantage of a temporal subdivision, with early release increasing v while bulk release increases q. The SARS-CoV-2 version of the CA model in *SI Appendix*, section C also illustrates this mechanism. To inform future iterations of this model, it would be useful to experimentally test the effect on v of mutations that affect viral reproduction at the cell level.

### Professional Immune Cells.

Finally, we consider how our model relates to aspects of the immune system not explicitly modeled. We have treated early infection as a competition between the invading virus and the structural tissue cells, assuming that professional immune cells only enter the stage a few days into a growing infection. In fact, tissue-resident immune cells, such as macrophages or dendritic cells, may be present at the infection site when a jumping virus arrives, and can mount strong antiviral responses (77). These responses may in turn affect the antiviral responses of the structural cells, as when TNF secreted by macrophages primes host cells to rapidly apoptose upon infection (78). The antiviral effect of professional immune cells may not change the dynamics qualitatively, but instead mainly affect parameter inference. If some infection foci are discovered in their infancy by sentinel immune cells, then this can be absorbed into q. If local growth is often halted by an immune cell, rather than an epithelial cell, then this can be represented as an adjustment to p. Inevitably, immune cells are subject to the same condition as the structural cells, that they must terminate an infection focus in its infancy in order to substantially curb viral growth.

While the model only purports to describe the early stage of infection, it is worth considering how one would model the transition to the subsequent stages of infection. Once a systemic immune reaction has been mounted, foci are now reproducing in an altered, less favorable environment. A simple extension to the model would be to track the total number $N_{n}$ of foci that have halted due to IFN and, once $N_{n}$ crosses a threshold, let the parameters change globally. For instance, q may drop, as many previously susceptible cells become refractory, while p increases due to positive feedback loops of cytokines.

In conclusion, this study offers a framework for thinking about viral spread in the host. The model presented is parsimonious, yet mechanistically detailed and empirically well-founded. Via the reproduction kernel, it can be flexibly adjusted to different pathogens or future experimental findings. Given the observation that the upper airways are perpetually teeming with viruses that fail to establish productive infection (79), a detailed understanding of the early stages of infection is key to preventing and controlling infectious diseases in the future. The growing evidence that viral spread in the respiratory tract is intrinsically spatial calls for modeling frameworks built around that fact. The model presented here is a step in that direction.

## Materials and Methods

### Introducing Eclipse Phase.

In this section, we derive the most detailed version of the model, including both a growth-free eclipse phase $a_{g}$ and a maximum age $a_{n}$ at which foci stop growing (Table 2). If the eclipse phase lasts until $a=a_{g}$, the probability per cell of being an IFN responder is p, and IFN is released at age $a_{n}$, solving

[18] $$\begin{array}{c} A\left(a_{n}\right)=\pi v^{2}{\left(a_{n}-a_{g}\right)}^{2}=\frac{1}{p}\Rightarrow a_{n}=a_{g}+\frac{1}{v\sqrt{\pi p}}. \end{array}$$

**Table 2.** Definitions of quantities used

Quantity name	Definition
a	Age of infection focus.
$a_{g}$	Age when focus starts growth.
$a_{n}$	Age when focus stops growth due to IFN.
q	Mean number of foci seeded by a cell.
p	Probability of a cell stopping local growth by IFN secretion.
v	Expansion speed of focus.
λ	Growth rate of system.
$\lambda_{0}$	Growth rate when p=0 ($a_{n}=\infty$).
k	$\sqrt{2\pi q}v$.
τ	$a-a_{g}$
$\tau_{n}$	$a_{n}-a_{g}$
α	$a_{g}\lambda_{0}$
β	$\tau_{n}\lambda_{0}$

The typical age at which a focus spawns a new focus of infection can be found similarly,

[19] $$\begin{array}{c} a_{q}=a_{g}+\frac{1}{v\sqrt{\pi q}}=a_{g}+\frac{\sqrt{2}}{k} \end{array}$$

with k still defined as Eq. **3**. Without IFN, the kernel is

[20] $$\begin{array}{c} b\left(a\right)=k^{2}\cdot \left(a-a_{g}\right)\cdot \theta \left(a-a_{g}\right). \end{array}$$

The Heaviside function is equivalent to introducing a lower bound in the integral of the Lotka-Euler equation. Thus, the growth rate in the absence of IFN, $\lambda_{0}$, solves

[21] $$\begin{array}{c} k^{2}\;\int_{a_{g}}^{\infty }\;e^{-\lambda_{0}a}\cdot \left(a-a_{g}\right)\;\;\text{d}a=1. \end{array}$$

This can be simplified by substituting a for $\tau \equiv a-a_{g}$, so we get

[22] $$\begin{array}{l} k^{2}\;e^{-\lambda_{0}a_{g}}\int_{0}^{\infty }\;e^{-\lambda_{0}\tau }\cdot \tau \;\text{d}\tau =\frac{k^{2}\;e^{-\lambda_{0}a_{g}}}{\lambda_{0}^{2}}=1\Rightarrow \\ k=\lambda_{0}e^{\lambda_{0}a_{g}/2}. \end{array}$$

Eq. **22** is a transcendental equation for $\lambda_{0}$ which can be written in terms of the Lambert W function (55),

[23] $$\begin{array}{c} \lambda_{0}=\frac{2}{a_{g}}W\left(\frac{a_{g}}{2}k\right)\;\mathrm{if}\;a_{g}>0,k\;\mathrm{if}\;a_{g}=0. \end{array}$$

Including IFN-activity at $a=a_{n}$, the Lotka-Euler equation gets an upper bound $\tau_{n}\equiv a_{n}-a_{g}$:

[24] $$\begin{array}{c} k^{2}\;e^{-\lambda a_{g}}\int_{0}^{\tau_{n}}\;e^{-\lambda \tau }\cdot \tau \;\;\text{d}\tau =k^{2}I\left(\lambda ;\;a_{g},a_{n}\right)=1, \end{array}$$

with

[25] $$\begin{array}{c} I\left(\lambda ;\;a_{g},a_{n}\right)\equiv e^{-\lambda a_{g}}\int_{0}^{\tau_{n}}\;e^{-\lambda \tau }\cdot \tau \;\;\text{d}\tau \\ =\frac{e^{-\lambda a_{g}}}{\lambda^{2}}\left(1-e^{-\lambda \tau_{n}}\left(1+\lambda \tau_{n}\right)\right). \end{array}$$

Now, using Eq. **22**, the Lotka-Euler equation becomes

[26] $$\begin{array}{c} k^{2}I\left(\lambda ;\;a_{g},a_{n}\right)=\lambda_{0}^{2}e^{\lambda_{0}a_{g}}\cdot \frac{e^{-\lambda a_{g}}}{\lambda^{2}}\left(1-e^{-\lambda \tau_{n}}\left(1+\lambda \tau_{n}\right)\right)=1. \end{array}$$

Notice that the jump probability q, which is the most difficult parameter to observe, is contained in k (Eq. **3**) but not in the integral and can thus be estimated by

[27] $$\begin{array}{c} q=\frac{1}{2\pi v^{2}\;I\left(\lambda ;\;a_{g},a_{n}\right)}. \end{array}$$

In the IFN-free case, the relation between q and $\lambda =\lambda_{0}$ becomes quite simple:

[28] $$\begin{array}{c} q=\frac{\lambda_{0}^{2}e^{\lambda_{0}a_{g}}}{2\pi v^{2}} \end{array}$$

Eq. **26** is quite messy, with many interdependent variables, so we next try to reduce it to the minimum number of independent variables. Defining $r\equiv \frac{\lambda }{\lambda_{0}}$, it can be written as

[29] $$\begin{array}{c} r^{2}=e^{\left(\lambda_{0}-\lambda \right)a_{g}}\left(1-e^{-\lambda \tau_{n}}\left(1+\lambda \tau_{n}\right)\right). \end{array}$$

Factoring out $\lambda_{0}$,

[30] $$\begin{array}{c} r^{2}=e^{\left(1-r\right)a_{g}\lambda_{0}}\left(1-e^{-\lambda_{0}r\tau_{n}}\left(1+\lambda_{0}r\tau_{n}\right)\right). \end{array}$$

Finally, defining two dimensionless quantities $\alpha \equiv \lambda_{0}a_{g}$ and $\beta \equiv \lambda_{0}\tau_{n}$ this condenses to

[31] $$\begin{array}{c} r^{2}=e^{(1-r)\alpha }\left(1-e^{-\beta r}\left(1+\beta r\right)\right). \end{array}$$

Thus, the relative effect r of introducing IFN depends on two independent variables α and β. The first is the eclipse before foci start growing, the second is the time window in which the focus is allowed to grow, both measured in terms of the characteristic time $1/\lambda_{0}$. Note that Eq. **31** is transcendental in λ and must be solved numerically.

We would like to be able to model the dynamics in terms of the more concrete biological parameter p, rather than $a_{n}$, which is more system-specific. To do this, define $\tau_{q}\equiv a_{q}-a_{g}$, similarly to $\tau_{n}$. Then

[32] $$\begin{array}{c} \tau_{n}=\frac{1}{v\sqrt{\pi p}},\;\tau_{q}=\frac{1}{v\sqrt{\pi q}}\Rightarrow \end{array}$$

[33] $$\begin{array}{c} \tau_{n}=\tau_{q}\sqrt{\frac{q}{p}}=\frac{\sqrt{2}}{k}\sqrt{\frac{q}{p}}. \end{array}$$

But since $\lambda_{0}=k\;e^{-\alpha /2}$ (Eq. **22**),

[34] $$\begin{array}{c} \beta =\lambda_{0}\tau_{n}=\sqrt{2}e^{-\alpha /2}\sqrt{\frac{q}{p}}. \end{array}$$

Thus, finally, we have shown that the relative significance r of IFN-signaling is uniquely determined by the combination of α (eclipse in units of the reproduction time-scale) and $\frac{q}{p}$ (the ratio of jump parameter to the IFN first-response rate). For this reason, the curves in Fig. 3*D* are general and not reliant on specific absolute parameter values.

## Supplementary Material

Appendix 01 (PDF)

## Data Availability

Simulation code and data are available at the public Github repository (80). AI statement: ChatGPT 5 and Claude Sonnet 4 aided in mathematical derivations and literature search. All mathematics in the article has been worked through by the authors and all cited sources have been read by the authors.
