# Supplementary material for: Bridging spatial scales in respiratory viral infection
Source: Proc Natl Acad Sci U S A. 2026 Oct 20;123(43):e2614436123. doi: 10.1073/pnas.2614436123 (PMC13715758; doi:10.1073/pnas.2614436123)
Supplement: Supplementary file 1 [file pnas.2614436123.sapp.pdf]

Proceedings of the
National Academy of Sciences
of the United States of America

Supplementary Information for

Bridging spatial scales in respiratory viral infection

Ulrik Hvid, Bjarke Frost Nielsen, Kim Sneppen

Kim Sneppen
ksneppen@gmail.com

This PDF file includes:

Figs. S1 to S7
Table S1
SI References

1. Supplementary material

A. Area contribution. The result that the mean area $\langle A \rangle$ is equal to $1/q$ in the exponential growth phase turns out to be independent of the specific reproduction kernel. Indeed, it follows solely from the relation between area and birth rate,

$$b(a) = q \cdot \frac{\mathrm{d}A}{\mathrm{d}a}, \tag{S1}$$

and the general Lotka–Euler equation,

$$\int_0^\infty b(a) e^{-\lambda a} \mathrm{d}a = q \int_0^\infty \mathrm{d}A \, e^{-\lambda a} = 1. \tag{S2}$$

Integrating by parts,

$$q \left[A(a) \, e^{-\lambda a} \Big|_0^\infty + \lambda \int_0^\infty A(a) \, e^{-\lambda a} \, \mathrm{d}a \right] = 1. \tag{S3}$$

Since $A(0) = 0$ and $A(a)$ grows slower than exponentially, the first term vanishes. Since $P(a) = \lambda e^{-\lambda a}$, the remaining integral is simply $\langle A \rangle$, giving

$$q \cdot \langle A \rangle = 1 \implies \langle A \rangle = \frac{1}{q}. \tag{S4}$$

B. Stochastic IFN activation. In the previous representation of the model, we made the simplifying assumption that IFN always gets activated at precisely the time when $A = A_n = 1/p$. In reality, activation is stochastic, and it is somewhat more realistic to treat it as a Poisson process. Then we can change the I of Eq. 27 to I_2, defined as

$$I_2(\lambda; a_g, a_n) = \int_{a_g}^\infty e^{-\lambda a} \cdot (a - a_g) \cdot e^{-A(a)/A_n} \mathrm{d}a = e^{-\lambda a_g} \int_0^\infty e^{-\lambda t} \cdot t \cdot e^{-\pi (v \ t)^2 p} \mathrm{d}t. \tag{S5}$$

with $t \equiv a - a_g$, $A(a) = \pi (v \ a)^2$ and $A_n = 1/p$. The solution is

$$I_2(\lambda; a_g, a_n) = e^{-\lambda a_g} \left[\frac{1}{2\pi v^2 p} - \frac{\lambda}{4\pi v^3 p^{3/2}} \, e^{\frac{\lambda^2}{4\pi v^2 p}} \operatorname{erfc}\left(\frac{\lambda}{2\sqrt{\pi} \, v \sqrt{p}} \right) \right] \tag{S6}$$

C. Solving the Lotka-Euler equation with a numerically derived kernel. In this section we implement a detailed cellular automaton (CA) model of the growth of a viral plaque, akin to previous models by Howat (2) and Lavigne (3). The purpose of this analysis is twofold. First, we aim to illustrate that, in the LEAP framework, the reproduction kernel $b(a)$ to be used in the LE equation (Eq. 6) need not be a simple analytical expression. It may instead be a data series for viral output over time from an infection focus. Such a data series may be derived from *in-vitro* measurement or, as in the present case, a mechanistic spatial model.

Second, we wish to explore the consequences of relaxing some assumptions of the piecewise linear kernels used in the main text (Fig. 1) and use known biological parameters more directly. There are, in particular, two important assumptions that we mean to challenge, regarding the early and late phases, respectively, of the life-cycle of an infection focus. First, the piecewise linear representation assumes that, after the release of the first virion, the front immediately starts progressing at its constant speed. In fact, it takes a finite amount of time to establish a front that can be regarded as continually expanding. The length of the transient depends on parameters that we have so far ignored, including the finite duration of the period in which the cell sheds virus. Second, we have assumed that, after the first cell releases IFN, viral shedding from the focus immediately halts. While this assumption does allow for a qualitative representation of the phenomenon that foci stop growing due to antiviral defenses, and that the reaction may be swift due to a paracrine amplification of IFN signaling (4–6), it is worth considering a more granular representation.

We first perform this analysis for IAV, and later implement a slightly more involved model to represent a SARS-CoV-2 focus.

Influenza A parameters. We represent the epithelial tissue as a square lattice, with the cells of the lattice representing epithelial surface cells. Each cell can be in one of the following states: T (susceptible target cell), E (virus-exposed, not yet virus-producing), V (virus shedding), N (IFN-secreting), A (refractory to viral infection), D (dead). The state transitions are illustrated in Fig. S3 and occur stochastically as Poisson processes.

The V state represents cells that are infected and currently shedding virus. On average, a cell occupies this state for a period τ_v. During this time, it produces virions at constant rate β/τ_v, producing on average β virions before transitioning to the state D (apoptosis/necrosis). For IAV, as a rough estimate, β is set to 100 infectious particles. Empirically, the actual number varies markedly between cells (7, 8), but 100 is the order of magnitude. Each virion is placed a distance l from the secreting cell, with l drawn from a Gaussian distribution of width σ, at a random angle (circular symmetry). The parameter σ is fitted to achieve a radial expansion speed of $1 d_c$/h (fig. S4A, table S1).

A T-cell hit by a virus immediately transitions to E, exposed, where it stays for an average duration τ_E. Most cells in the E state transition to V, but a fraction p transition to N, immediately secreting IFN to warn other cells in the system. Every time a cell reaches the N-state, this increases the IFN-concentration n in the system by $\Delta_n = 1$, effectively a choice of unit. As in (2), we assume that IFN immediately spreads homogeneously throughout the local tissue. This is supported by experimental observations that the IFN signal propagates faster than the infection front (9).

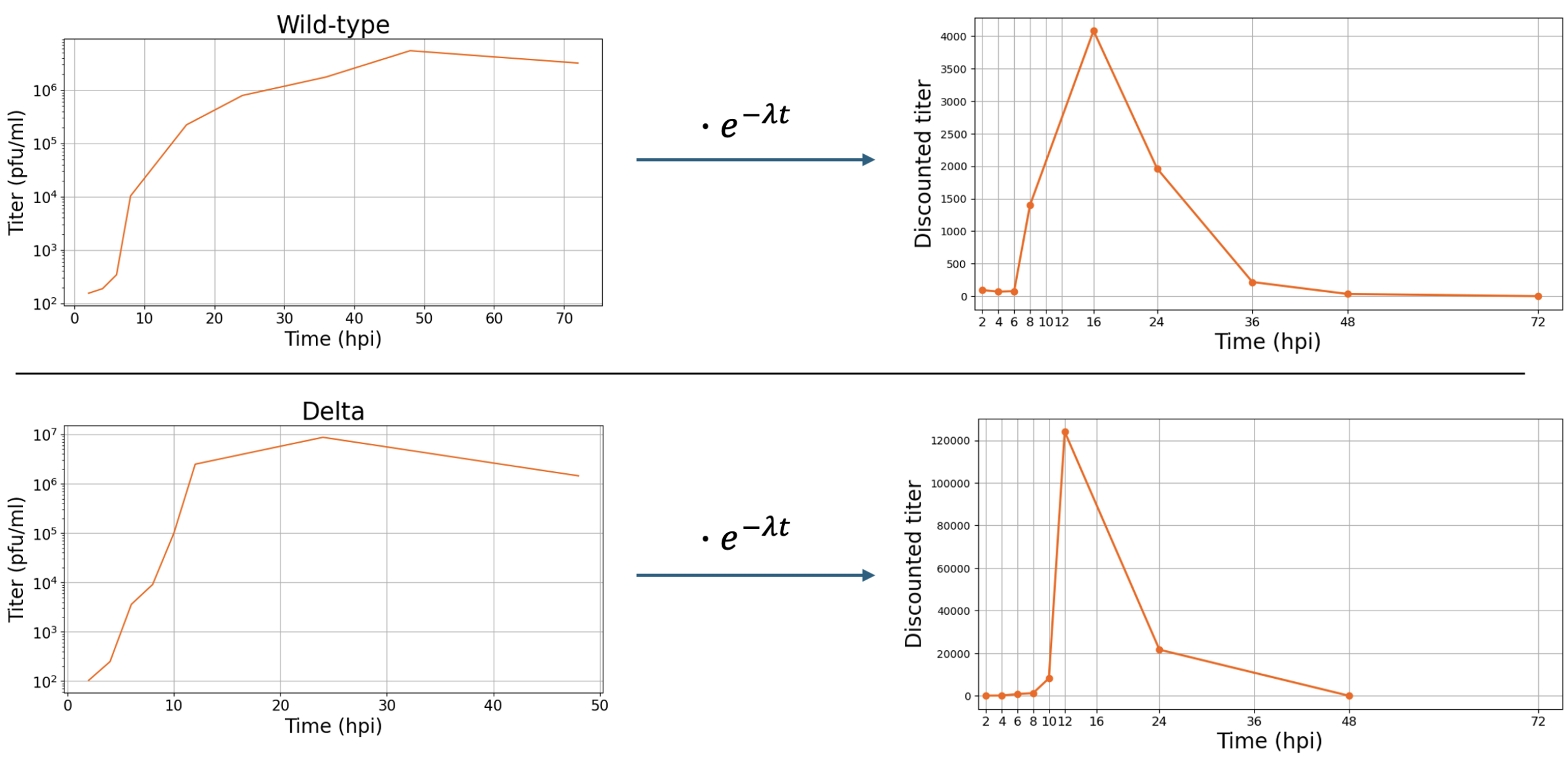

Fig. S1. Applying exponential discount to one-step growth curves yields a_g estimate Left: Replotting of one-step growth curves from (1). These curves are titer measurements from cultures infected at high MOI, such that they represent the output of just one viral generation. Data collected from figures in (1) with graphreader.com. Right: Discounting late releases by multiplying by an exponential function with $\lambda = 0.25\ \mathrm{h}^{-1}$ yields shapes with clear peaks at 16 and 12 hpi respectively. These are our estimates of a_g for SARS-CoV-2 WT and Delta.

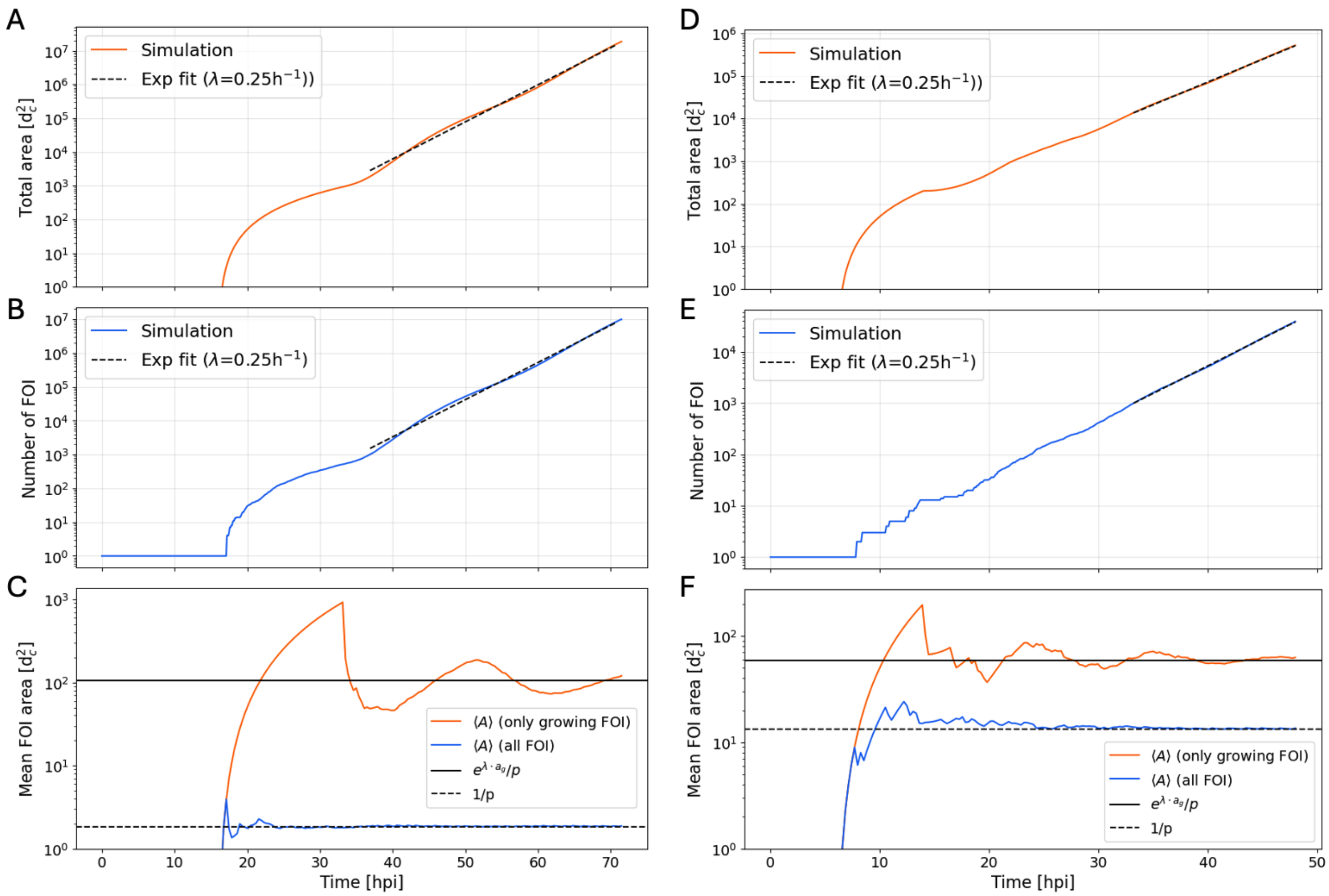

Fig. S2. Stochastic simulation of the reproduction kernel shows transient and exponential phase. Simulation using the parameter values in Table 1 with **A, B, C** using SARS-CoV-2 parameters and **D, E, F** using parameters for IAV. Both total area **A** and number of foci **B** show a transient period with oscillations on the timescale of a_g h, before settling into steady exponential growth with $\lambda = 0.25$/h as predicted analytically. Panel **C** shows the mean value across all foci (blue) and all growing foci (red) respectively. As non-growing foci will be invisible or tiny in a measurement using fluorescent proteins, the red curve is more representative of what might actually be observed, i.e., a typical focus size of ≈ 100 cells.

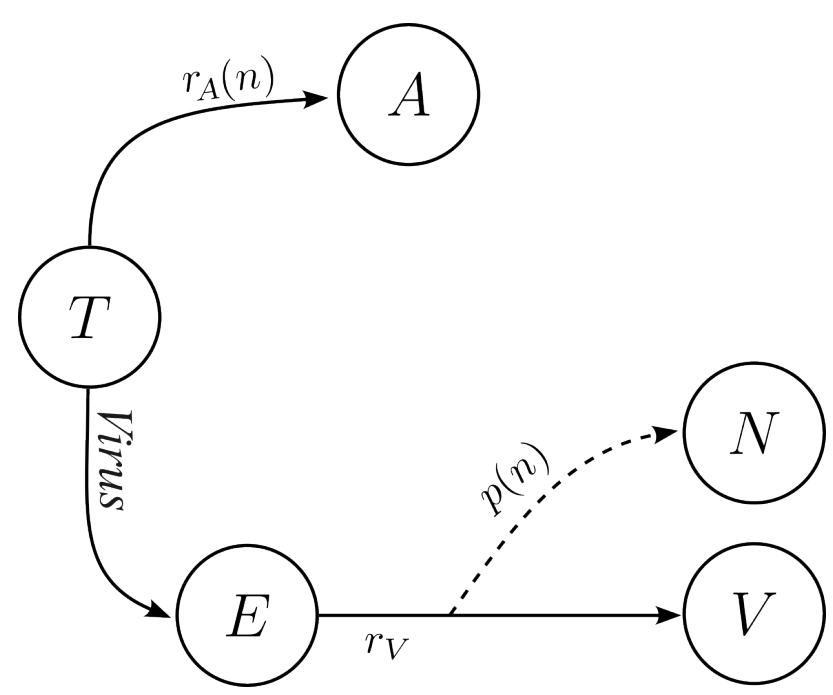

Fig. S3. State transitions in the CA model. In the cellular automaton model of a growing plaque, susceptible target cells (T) may transition to the antiviral state (A) when exposed to interferon n, or to the exposed state E when exposed to virus. Most E-cells transition to virus producing, but an n-dependent fraction transition to the IFN producing state N.

IFN has two effects in the model. First, cells in the T-state respond to IFN by transitioning to the antiviral state A. They transition at a rate governed by the Hill equation:

$$r_A = \frac{r_{A,max}\ n}{n + n_0}. \tag{S7}$$

Second, IFN initiates a self-amplification loop, as higher n increases the probability that future E-cells transition to N instead of V. Therefore, we let p depend similarly on the IFN concentration:

$$p(n) = p_0 + \frac{(p_{max} - p_0)\ n}{n + n_0}, \tag{S8}$$

using the same half-max concentration, n_0. We call p_0 the "first responder probability" and p_{max} the "second responder probability," borrowing terminology from van Eyndhoven et al. (4). The former probability can be estimated experimentally by infecting isolated cells and measuring the fraction that secrete IFN. For IAV, $p_0 = 0.5\%$ (10). As the local tissue saturates with IFN, p increases to the second responder probability p_{max} which we set to 5%. We set n_0 to 10, meaning that when $\gg 10$ cells have secreted IFN in a short time span, the local tissue is maximally alarmed. Finally, IFN decays at rate $\theta_n = 0.1\text{h}^{-1}$. The parameters related to dynamics downstream of the first IFN-response, p_{max}, n_0 and θ_n, are difficult to estimate from literature, but we show that results are relatively insensitive to these values.

The IFN amplification loop, in which paracrine IFN signaling can increase the fraction of cells that secrete IFN in turn, is well-established (4–6). Experiments have been performed to disentangle the role of the rare first responder cells from that of the second responders by, for instance, chemically blocking IFN sensing with Ruxolitinib (6) or plating cells at low density (5), restricting paracrine amplification. In the CA model, we let the first responder probability p_0 vary with the virus type (0.5% for IAV and 0.01% for SARS-CoV-2, as in Table 1) but let the second responder probability $p_{max} = 5\%$ for both viruses.

First, to demonstrate the emergence of a fixed radial speed, we run the simulation with $p_0 = 0$, ensuring that no IFN is produced in the system. We define the radius of the plaque to be the mean distance of cells in E and V states from the seed cell. As mentioned, σ was adjusted to make sure that, after a transient, the radius increases at a rate of $1d_c$/h. As Fig. S4A shows, the radius increases in a slightly convex fashion for the first few generations. Thus, even though the slope is eventually correct, the curve is somewhat delayed compared to the line that starts at $a = a_g = 6$h, corresponding to kernel (ii) in the main text. Fig. S4B shows that the viral shedding rate, too, converges to a linear increase, as we expect, given that all viral output comes from the periphery of the focus of infection.

Finally, we introduce IFN. Fig. S4C shows an example run of the CA model. IFN secretion typically sets in after a couple of generations, gradually curbing the spread of the focus.

Now that we have a complete representation of a virus-producing focus, we are ready to apply the LE equation to solve for the jump rate q that gives the correct exponential growth rate. The following procedure translates the measured viral output from the focus into a reproduction kernel. Denote the total viral production rate from the focus at age a by $F(a)$. Let $p_v \ll 1$ be the probability per virion of jumping and establishing a plaque in a distant region of the respiratory tract. Then the reproduction kernel is $b(a) = F(a) \cdot p_v$. We are interested in the number of successful jumps per cell, $q = p_v \beta$. Then,

$$b(a) = F(a) \cdot q/\beta. \tag{S9}$$

Since the simulation produces a value of $F(a)$ at each time point a, we can now perform a numerical integral to find the q value that satisfies the LE equation:

$$\int_0^{t_f} b(a) \cdot \mathrm{e}^{-\lambda a} \mathrm{d}a = \int_0^{\infty} F(a) \cdot \frac{q}{\beta} \cdot \mathrm{e}^{-\lambda a} \mathrm{d}a = 1 \tag{S10}$$

Notice that, even though β is only known very roughly, this has little consequence in eq. S10, since $F(a)$ is proportional to β which then cancels out (neglecting a slight effect of β on the propagation speed, which in turn is canceled in the σ-fit). In contrast, an estimated jump probability per virion p_v would depend on the assumed β.

Since state transitions are stochastic, $F(a)$ may vary considerably between runs, and we take a mean of several runs and use that to solve eq. S10 for q. The result is shown in fig. S5. Panel A clearly shows that, in this more detailed model, IFN is still able to contain the spread of the plaque, but it allows for the release of large amounts of virus after the first cell secretes IFN. The IFN cascade is, with these parameters, not nearly strong enough to stop plaque growth immediately. Still, even though viruses released after the onset of IFN signaling make up a majority of the total viral yield from the focus (panel A), this majority actually has relatively little effect on the integrand of the LE equation shown in panel B. Solving the LE equation S10 using the mean curve as $F(a)$, we obtain $q = 0.19$, meaning that about a fifth of IAV infected cells seed a new plaque.

As previously mentioned, the parameters regarding the effect of IFN are very roughly estimated. As a sensitivity check, we re-compute q in three extreme settings of the IFN dynamics.

1: No IFN signaling ($p_0 = 0$). In this case $q = 0.19$, indistinguishable from the intermediate case represented in Fig. S5.

2: IFN signaling is substantially strengthened ($\Delta_n = 3$, $p_{max} = 0.2$) relative to the base parameters in table S1. Here, $q = 0.21$, marginally increased from the base parameters. This illustrates the relative insensitivity to parameters describing dynamics downstream of the first IFN response.

3: IFN signaling immediately halts all viral release, including from currently shedding cells. Here, we find $q = 0.38$.

Parameter	Description	Value	Unit	Source
		Grid & Time		
L	Grid side length	500	cells	—
Δt	Time step	0.1	h	—
		Influenza A		
τ_E	Rate $E \to V$	6	h	(11)
τ_V	Mean V-cell lifetime	6	h	(11)
β	Burst size	100	virions cell^{-1}	(7, 8)
σ	Viral spread std (2D Gaussian)	1.35	d_c	Fitted to expansion speed
p_0	First responder probability	5×10^{-3}	d_c^2	(10)
$p_{\max}$	Second responder probability	5×10^{-2}	d_c^2	(4–6)
Δ_n	IFN released per N cell	1	—	Choice of unit
n_0	Half-saturation n	10	—	—
θ_n	IFN decay rate	$1/10$	h^{-1}	—
$r_{A,\max}$	Max rate $T \to A$	$1/10$	h^{-1}	(12)
		SARS-CoV-2		
τ_E	Rate $E \to V_1$	6	h	(1), Fig. S1
p_0	First responder probability	10^{-4}	d_c^2	(13)
$p_{\max}$	Second responder probability	5×10^{-2}	d_c^2	(4–6)
τ_{V1}	Lifetime in early productive phase	6	h	(1), Fig. S1
τ_{V2}	Lifetime in late productive phase	6	h	(1), Fig. S1
β_1	Burst size in early phase	5	virions cell^{-1}	(14)
β_2	Burst size in late phase	500	virions cell^{-1}	(14)
σ	Viral spread std (2D Gaussian)	1.75	cells	Fitted to expansion speed

Table S1. Parameters for cellular automaton model

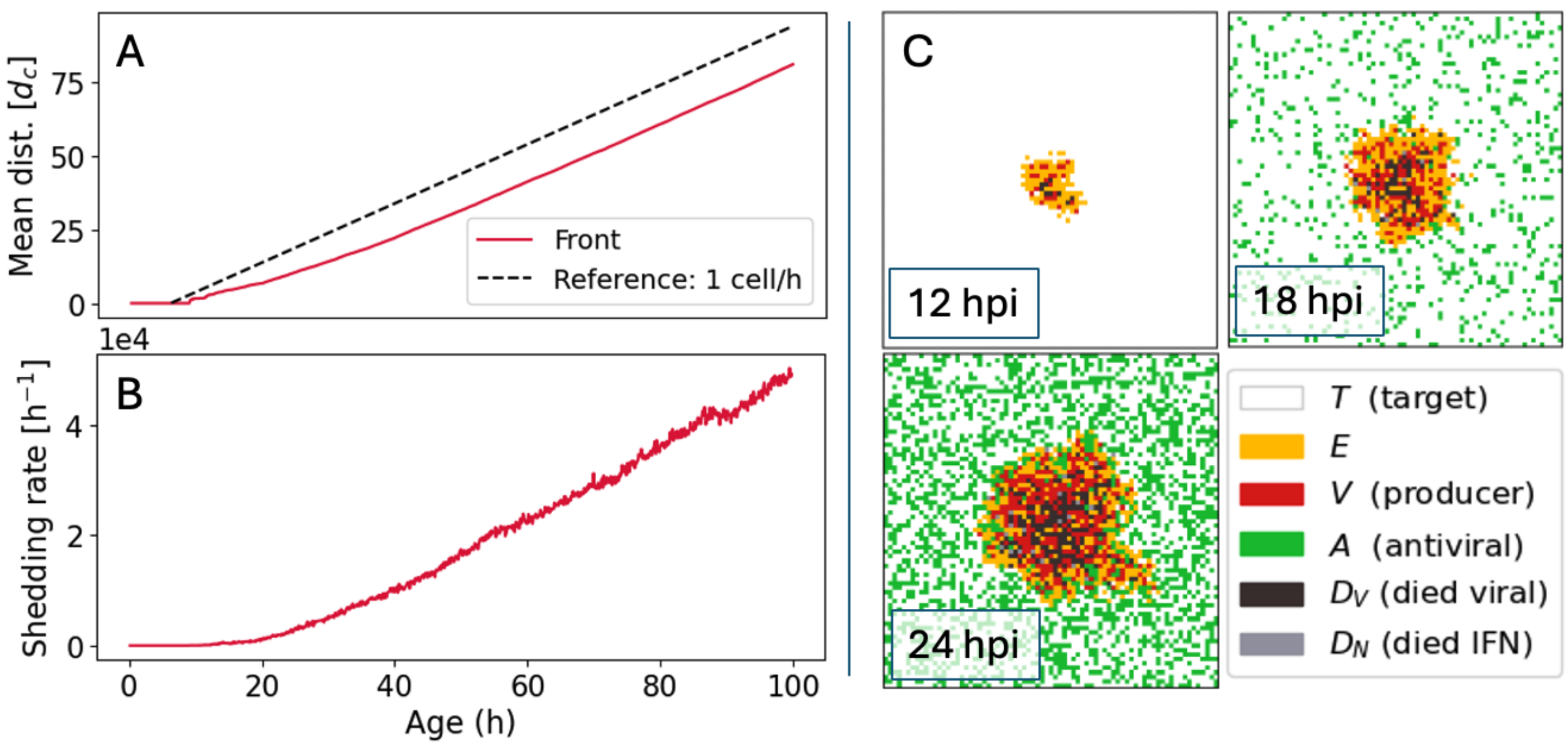

Fig. S4. Cellular automaton model produces linearly growing plaque and shedding rate, after a transient. **A** The radius of the simulated plaque, defined as the mean distance of all E and V cells from the seed cell. The parameter σ was tuned to produce an asymptotic expansion speed of 1 d_c/h (Table S1). **B** The shedding rate, too, asymptotes to a linear increase, as expected, since all shedding happens from the front, and cells behind the front are dead. **C** An example run, with IAV parameters. In the early phase, the viral focus spread unrestricted. At some point, a first responder releases IFN, triggering the first few cells to go to the A-state, and triggering a handful of second responders to boost the signal. This leads to an increasing concentration of refractory cells, stopping the growing plaque.

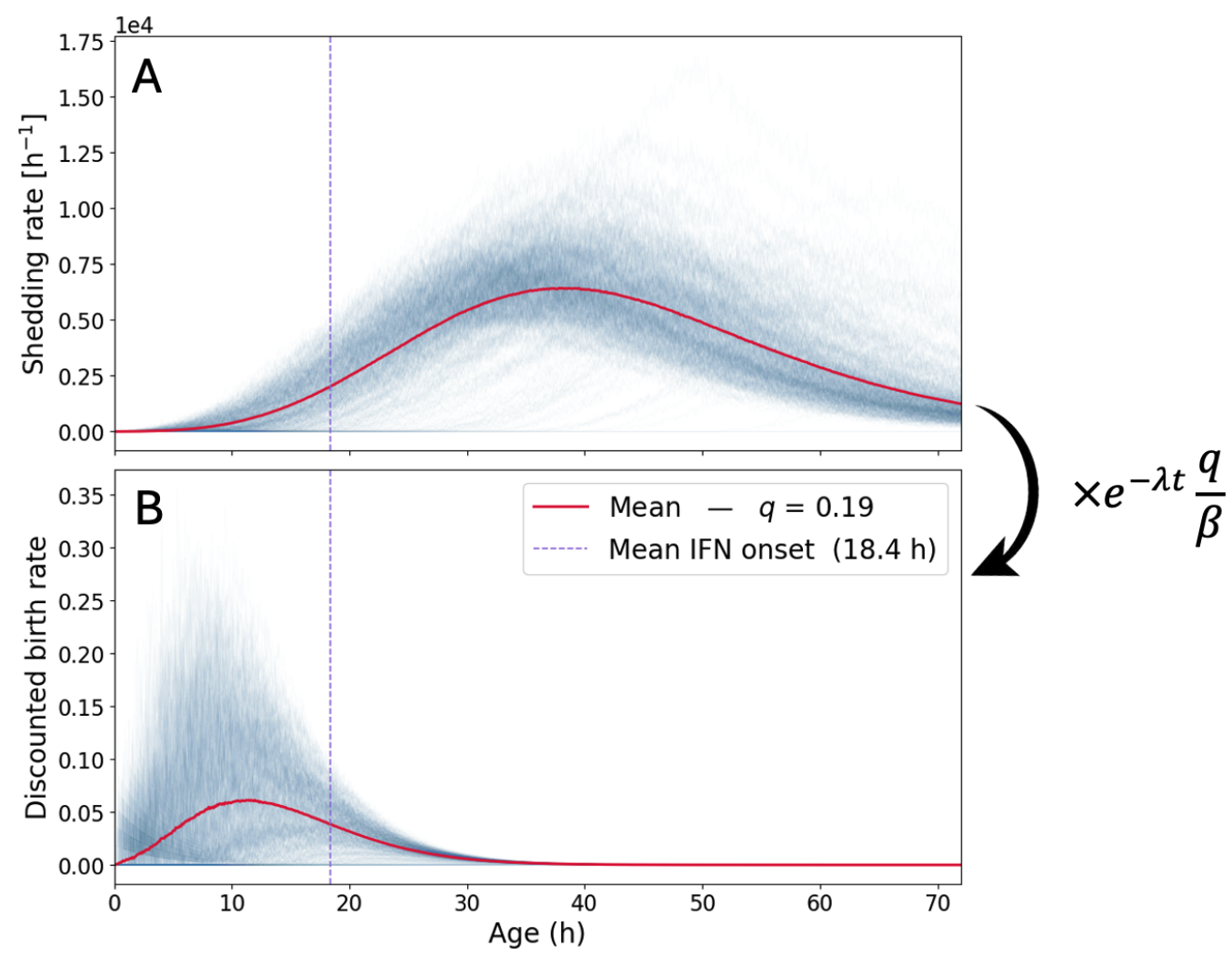

Fig. S5. Most viral particles are released after the onset of IFN, but they have little effect on exponential growth. A The figure shows shedding rate over time for 500 runs (blue, transparent) as well as the mean (red) with IAV parameters. The mean curve is inserted as $F(a)$ into eq. S10 to solve for q. **B** Having solved for q, we calculate the discounted and normalized growth rate (the integrand in eq. S10) for the 500 runs and the mean, whose area, by construction, is equal to 1.

The difference between no IFN signaling (case 1) and an infinitely strong IFN cascade (case 3) is a factor of 2 in the estimated q. This conclusion mirrors the finding in the main text that IAV exists in a range where the first responder probability has an influence on the exponential growth rate, meaning that IAV can grow faster by increasing its capability to antagonize IFN. The referenced Github repository contains the code for running this test (use `mode = "no_IFN"`, `"strong_IFN"` or `"step_IFN"`).

SARS-CoV-2 parameters. Before repeating the analysis for SARS-CoV-2, we point out again two important differences between this virus and IAV in terms of viral kinetics. First, WT SARS-CoV-2 appears to have a complex one-step growth curve (the estimated viral kinetics of an individual cell), with a viral shedding rate that increases rapidly between 6 hpi and 16 hpi (Fig. S1). To represent this, we model two consecutive states of viral shedding, V_1 and V_2, each lasting 6h. Then we set the shedding rate in V_1 to be 1% of that in V_2. Then, the few early releases at ≈ 6 hpi set the plaque expansion speed, while the vastly more numerous later releases at $\approx 12 - 18$ hpi allow for many more jumps per cell, enough to overcome the exponential discounting.

Second, a distinguishing feature of SARS-CoV-2 is its ability to avoid detection and restrict cytokine signaling (15). Therefore, as in the main text, we set $p_0 = 10^{-4}$. While it is well documented that SARS-CoV-2 is effective at delaying IFN response, implying a low first responder probability, we have not been able to derive a second responder probability p_{max}, and we set it to the same value as for IAV. As we will see soon, this has no influence on the estimated q. Nor have we managed to find precise measurements of the viral yield per cell, but based on a rough estimate (14) we set it to ≈ 500. Specifically, in the two-rate representation of viral-shedding described above, the two burst sizes are $\beta_1 = 5$ and $\beta_2 = 500$. As explained in the previous section, the chosen burst size has little effect on the q-estimate.

The parameters in table S1 yield the dynamics seen in fig. S6. In terms of timing and plaque size, these dynamics correspond roughly to the measurements in ALI culture in (13).

For SARS-CoV-2, the numerically derived kernel gives $q = 0.70$, a $\approx 40\%$ increase from the analytical derivation in the main text. Similar to our conclusion from the analytical kernels, SARS-CoV-2 is comfortably within the range where IFN has no effect on the exponential growth rate. As IFN signaling is only activated after the discounted birth rate has gone to 0, making the IFN cascade stronger or weaker has no detectable effect on q.

Comparing simulation and analytical kernels. Though the estimates of q shift somewhat between the analytical reproduction kernels chosen in the main text and the ones derived from a CA model above, the conclusion remains robust: IAV appears to differ from WT SARS-CoV-2 in terms of the role played by IFN. The latter virus is able to suppress IFN signaling in a focus of infection for so long that, when it finally happens, the discounted birth rate of the focus in question has long since decayed (fig. S6, and the infection is now dominated by its offspring. For IAV, which is less effective at antagonizing antiviral activity, IFN secretion starts before the LE integrand has gone to zero (fig. S5), meaning that IAV may benefit from further enhancing its immune-antagonizing capabilities.

The logic of the LE equation implies that early focus dynamics determine the growth rate, while the behavior of older foci is of vanishing importance. Consequently, the difference in the q-estimates from the analytical kernels (Fig. 1B) and the CA model, respectively, primarily stems from assumptions regarding the early growth of the focus. The analytical kernels assume that, the moment the focus of infection starts growing, it immediately achieves a linearly increasing birth rate $b(a)$. The cellular automaton can represent the convex shape of $b(a)$ that results from the fact that a continually expanding front only becomes an appropriate description in the second viral generation when many cells are producing viral offspring simultaneously. The analytical kernel gives the virus an advantage by, in a sense, skipping a generation, and the LE equation compensates by predicting a lower q value, retaining the correct exponential growth rate.

D. Domain of effectiveness of p. We can arrive at an approximate expression for the ratio q/p above which IFN becomes ineffectual, as follows. First, we rewrite Eq. 31 as:

$$e^{-x}(1+x) = 1 - r^2 e^{-(1-r)\alpha} \quad \text{[S11]}$$

with $x \equiv \beta r = \lambda \tau_n$ which is essentially the number of e-folds by which the focus has grown before being halted by IFN. This function has maximal curvature at $x = 2$, signifying the steepest diminishment of returns. We thus expand the above expression around $x = 2$ find:

$$1 - r^2 e^{-(1-r)\alpha} \approx \frac{3}{e^2} - \frac{2}{e^2}(x-2) \quad \text{[S12]}$$

solving for x yields:

$$x \approx \frac{7 - e^2}{2} + \frac{e^2}{2} r^2 e^{-(1-r)\alpha}. \quad \text{[S13]}$$

One may keep the first (constant) term for higher accuracy, but in our case it will always be dwarfed by the second term, and we thus write:

$$x \approx \frac{e^2}{2} r^2 e^{-(1-r)\alpha}. \quad \text{[S14]}$$

We then reinstate the biologically intuitive parameters, p, a_g, and λ_0 (using Eq. 34) and find:

$$\frac{p}{q} \approx 8e^{-4} r^{-2} e^{(1-2r)\lambda_0 a_g}. \quad \text{[S15]}$$

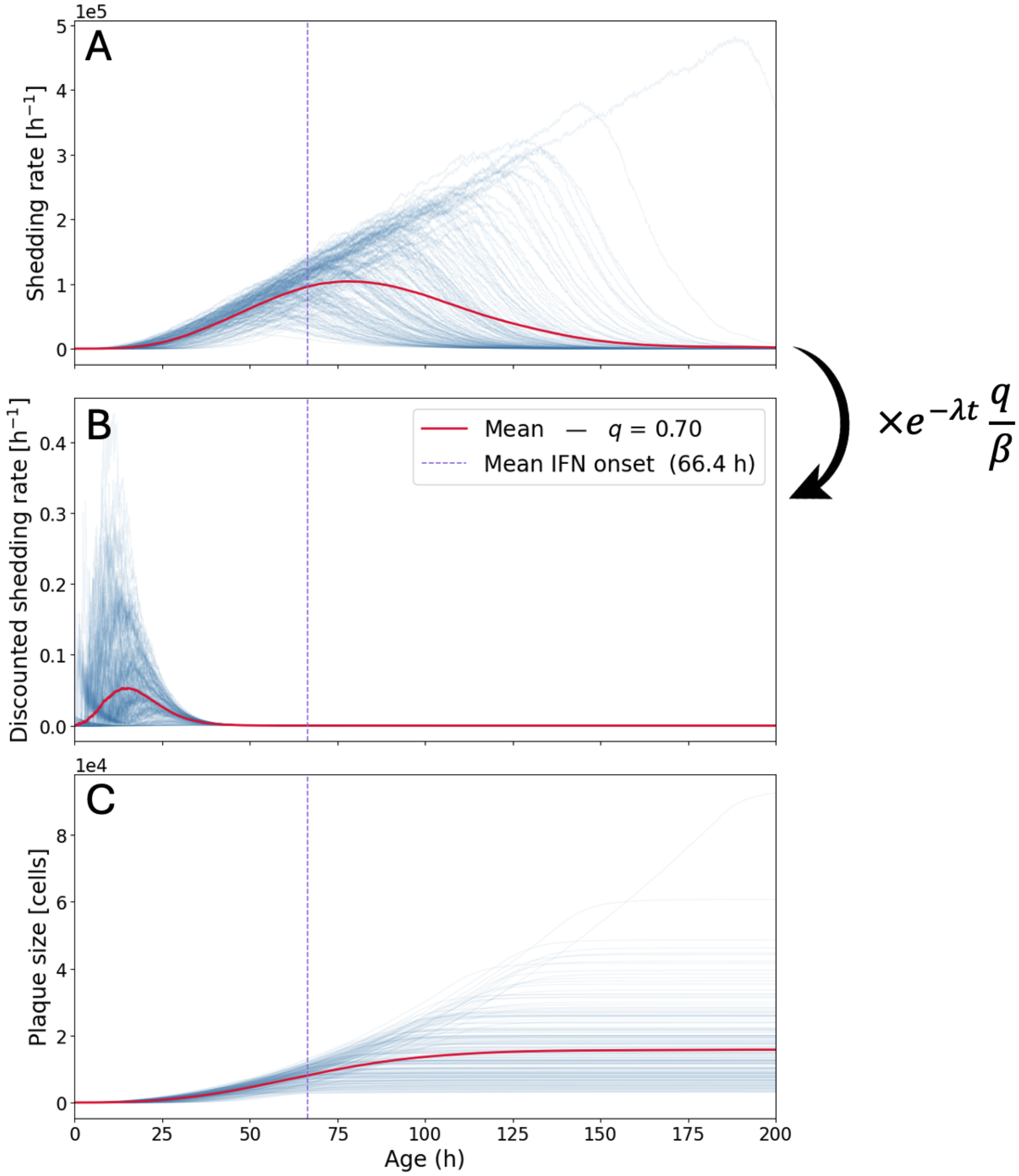

Fig. S6. With SARS-CoV-2 parameters, viruses released after IFN onset are inconsequential for growth rate. A and **B** are similar to the equivalent panels in Fig. S5, but using SARS-CoV-2 parameters. In this case, the discounted shedding rate has entirely vanished at the average onset time of IFN, implying that IFN signaling has no effect on SARS-CoV-2 growth rate. **C** Since the first responder probability p_0 for SARS-CoV-2 was estimated based on the typical maximum sizes of plaques in ALI cultures, we show the plaque sizes over time, for comparison with Becker et al. (13). Plaque size is defined here as the number of cells that are not T or A, ie. have been infected by a virus. The timing and height of the curves roughly agree with those in (13), though the experimental curves eventually decrease, as they count cells positive for SARS-CoV-2 protein, which will eventually decay in dead cells.

To further simplify the expression, we take the approximation $r \approx 1$ (strongly diminished returns) and end up with

$$\frac{p}{q} \approx 8e^{-4}e^{-\lambda_0 a_g}. \quad \text{[S16]}$$

Fig. S7 shows a heatmap of λ/λ_0 as a function of q/p and a_g. We include the curve corresponding to eq. S16 and see that it reasonably well separates the area in which the λ/λ_0 changes with the parameters, and the one in which it does not.

References

1. N Khandelwal, et al., Studies on growth characteristics and cross-neutralization of wild-type and delta SARS-CoV-2 from hisar (india). *Front. Cell. Infect. Microbiol.* **11** (2021).
2. TJ Howat, C Barreca, P O'Hare, JR Gog, BT Grenfell, Modelling dynamics of the type i interferon response toin vitroviral infection. *J. The Royal Soc. Interface* **3**, 699–709 (2006).
3. G Michael Lavigne, H Russell, B Sherry, R Ke, Autocrine and paracrine interferon signalling as 'ring vaccination' and 'contact tracing' strategies to suppress virus infection in a host. *Proc. Royal Soc. B: Biol. Sci.* **288**, 20203002 (2021).
4. LC Van Eyndhoven, A Singh, J Tel, Decoding the dynamics of multilayered stochastic antiviral IFN-i responses. *Trends Immunol.* **42**, 824–839 (2021).
5. S Patil, et al., Single-cell analysis shows that paracrine signaling by first responder cells shapes the interferon-β response to viral infection. *Sci. Signal.* **8** (2015).
6. EA Thayer, et al., Single-cell heterogeneity in interferon induction potential is heritable and governed by variation in cell state (bioRxiv) (2025) Preprint.
7. GK Zath, et al., Influenza A viral burst size from thousands of infected single cells using droplet quantitative pcr (dqpcr). *PLOS Pathog.* **20**, e1012257 (2024).
8. FS Heldt, SY Kupke, S Dorl, U Reichl, T Frensing, Single-cell analysis and stochastic modelling unveil large cell-to-cell variability in influenza A virus infection. *Nat. Commun.* **6** (2015).
9. EA Voigt, A Swick, J Yin, Rapid induction and persistence of paracrine-induced cellular antiviral states arrest viral infection spread in a549 cells. *Virology* **496**, 59–66 (2016).
10. AB Russell, E Elshina, JR Kowalsky, AJW te Velthuis, JD Bloom, Single-cell virus sequencing of influenza infections that trigger innate immunity. *J. Virol.* **93** (2019).
11. T Frensing, et al., Influenza virus intracellular replication dynamics, release kinetics, and particle morphology during propagation in mdck cells. *Appl. Microbiol. Biotechnol.* **100**, 7181–7192 (2016).
12. U Rand, et al., Multi-layered stochasticity and paracrine signal propagation shape the type-i interferon response. *Mol. Syst. Biol.* **8** (2012).
13. ME Becker, et al., Live imaging of airway epithelium reveals that mucociliary clearance modulates SARS-CoV-2 spread. *Nat. Commun.* **15** (2024).
14. YM Bar-On, A Flamholz, R Phillips, R Milo, SARS-CoV-2 (COVID-19) by the numbers. *eLife* **9** (2020).
15. CF Hatton, et al., Delayed induction of type i and iii interferons mediates nasal epithelial cell permissiveness to SARS-CoV-2. *Nat. Commun.* **12** (2021).

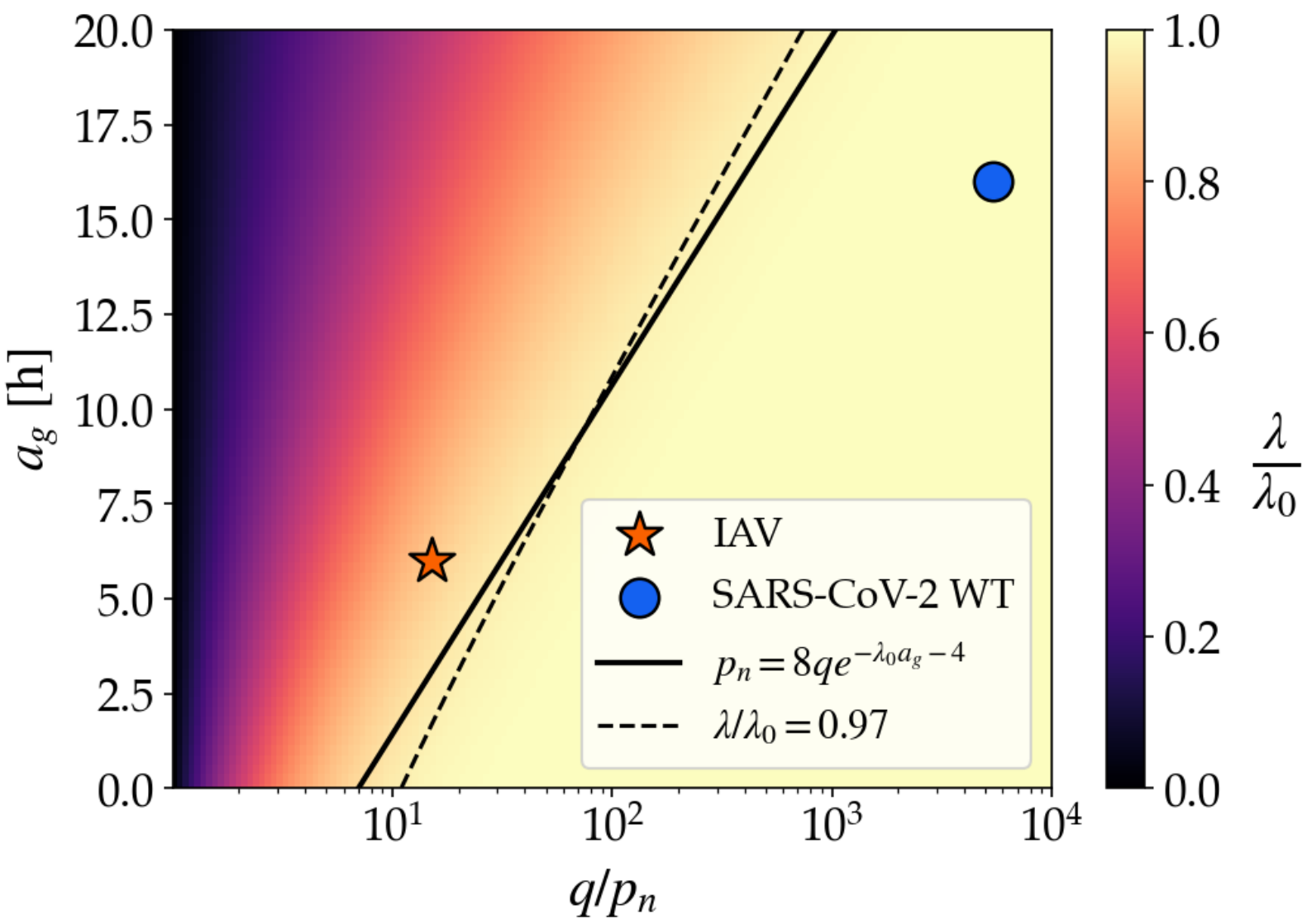

Fig. S7. A simple approximation for the domain of effectiveness of p. Heatmap of λ/λ_0 as a function of q/p and a_g. The quantity $r \equiv \lambda/\lambda_0$ is isolated in Eq. 31. Also shown are the curve representing Eq. S16 and a contour curve where $r = 0.97$. Eq. S16 separates the area in which r changes with parameters from the area in which it does not.
